# Cost-effectiveness of screening, decolonisation and isolation strategies for carbapenem-resistant Enterobacterales and methicillin-resistant *Staphylococcus aureus* infections in hospitals: a sex-stratified mathematical modelling study

**DOI:** 10.1016/j.lana.2025.101019

**Published:** 2025-02-15

**Authors:** Kasim Allel, Patricia Garcia, Anne Peters, Jose Munita, Eduardo A. Undurraga, Laith Yakob

**Affiliations:** a Nuffield Department of Primary Care Health Sciences, University of Oxford, Oxfordshire, United Kingdom; b School of Medicine, Pontificia Universidad Católica de Chile, Santiago, Chile; c Department of Disease Control, London School of Hygiene and Tropical Medicine, London, United Kingdom; d Genomics and Resistant Microbes (GeRM), Facultad de Medicina Clínica Alemana, Instituto de Ciencias e Innovación en Medicina (ICIM), Universidad del Desarrollo, Santiago, Chile; e School of Government, Pontificia Universidad Católica de Chile, Santiago, Chile; f Research Center for Integrated Disaster Risk Management (CIGIDEN), Chile

**Keywords:** Mathematical modelling, Antibiotic resistance, Transmission dynamics, Interventions, Cost-effectiveness

## Abstract

**Background:**

Methicillin-resistant *Staphylococcus aureus* (MRSA) and carbapenem-resistant Enterobacterales (CRE) impose the greatest burden among critical bacterial pathogens. Evidence for sex differences among antibiotic resistant bacterial infections is increasing but a focus on policy implications is needed. We assessed impact of CRE/MRSA on excess length of hospital stay, intensive care unit admission, and mortality by sex from a retrospective cohort study (n = 873) of patients in three Chilean hospitals, 2018–2021.

**Methods:**

We used inverse-probability weighting combined with descriptive, logistic, and competing-risks analyses. We developed a sex-stratified deterministic compartmental model to analyse hospital transmission dynamics and the cost-effectiveness of nine interventions. We compared interventions based on the incremental cost-effectiveness ratio (ICER) per quality-adjusted life year (QALY) gained and estimated net benefits.

**Findings:**

The adjusted odds of women acquiring CRE and MRSA were 0.44 (0.28–0.70; p = 0.0013) and 0.73 (95% CI = 0.48–1.01; p = 0.050), respectively. Competing-risk models indicated higher mortality rates among women, compared to men. Mathematical model projections showed that pre-emptive isolation across all newly admitted high-risk men was the most cost-effective intervention (ICER = $1366/QALY and $1083/QALY for CRE and MRSA, respectively). Chromogenic agar coupled with MRSA decolonisation was the second most cost-effective intervention ($2099/QALY), followed by screening plus isolation or pre-emptive isolation strategies (ICER ranged between $2411/QALY and $4216/QALY across CRE and MRSA models). Probabilistic sensitivity analysis showed that strategies were ICER < willingness-to-pay in 80% of simulations, except for testing plus digestive decolonisation for CRE. At a 20% national hospital coverage at least $12.2 million could be saved.

**Interpretation:**

Our model suggests that targeted infection control strategies would effectively address rising CRE and MRSA infections. Maximising health-economic gains may be achieved by focusing on control measures for men as primary drivers for transmission, thereby reducing the disproportionate disease burden borne by women.

**Funding:**

Agencia Nacional de Investigación y Desarrollo 10.13039/501100020884ANID, Chile.

**Research in context**

**Evidence before this study**

Despite reports of differences in antibiotic resistance impacts between biological sexes, evidence remains sparse, particularly in subgroup-specific intervention effects and policymaking. We searched the literature for studies evaluating the cost-effectiveness of pharmaceutical and non-pharmaceutical interventions aimed at reducing, monitoring and controlling *S. aureus* (MRSA) and carbapenem-resistant Enterobacterales (CRE) in patients. Articles published until the 1st of March 2024 were explored using EconLit, EMBASE and PubMed. Our literature search was guided by four primary themes: (1) ‘Interventions for CRE/MRSA, (2) ‘Hospital’, (3) ‘Cost-effectiveness and economic evaluation’, and (4) ‘Population type/sub-groups’. We did not use language restrictions. We found that most studies have established the effectiveness of various interventions to control the spread of MRSA and CRE, such as screening, isolation, and decolonisation. These interventions are generally implemented across whole hospital populations, overlooking established associations with individual-level risk factors, such as biological sex, and specific behaviours or characteristics that predispose men and women to CRE/MRSA acquisition.

**Added value of this study**

Our study enhances existing knowledge by detailing how sex differences influence the outcomes of CRE/MRSA infections in Chilean hospitals, highlighting an underexplored area in infection control. By integrating these differences into our mathematical model, our findings offer a novel perspective on optimising intervention strategies. Specifically, the targeted approach for men, who are at higher risk of acquiring these infections, not only improves outcomes by increasing quality-adjusted life years (QALYs) but also maximizes health-economic benefits. This strategic focus enables more effective resource allocation and better prioritisation in health policy, providing a framework for scaling interventions according to cost-effectiveness.

**Implications of all the available evidence**

This study's findings are pivotal for shaping public health policies and refining hospital infection control measures. Implementing strategies customised to patient risk profiles, health impacts, transmission dynamics, and intervention responses across diverse patient cohorts promises to amplify the efficacy of infection control efforts, thereby enhancing net monetary benefits nationally.

## Introduction

Antibiotic-resistant bacteria (ARB) represent a pressing global health challenge,^1^ with sociodemographic and anthropogenic factors playing key roles in their spread.^2^ Among the critical bacterial pathogens, methicillin-resistant *Staphylococcus aureus* (MRSA) and carbapenem-resistant Enterobacterales (CRE) impose the greatest burden. In South America, MRSA and CRE comprise more than half of all reported ARB cases, contributing to 56.3 (40.2–76.3) age-standardised deaths per 100,000 individuals.^3^ Our study was conducted in Chile where one-third of *S. aureus* infections and one-half of Enterobacterales infections were recently reported to be caused by resistant pathogens, contributing to about $10,300 in excess hospital costs per patient.^4^ Current guidelines in Chile's National Action Plan includes protocols for infection control and antibiotic stewardship programmes.^5^ However, the limited information on the costs and cost-effectiveness of these interventions to reduce antibiotic resistance hinders efficient resource allocation.^6^

Some non-pharmaceutical interventions have emerged as highly cost-effective.^7^ Decolonisation (e.g., using topical agents or selective digestive decontamination),^8^^,^^9^ diagnostics (e.g., chromogenic agar, polymerase chain reaction),^10^^,^^11^ and isolation via contact precaution (e.g., use of gowns and gloves)^12^ have been popular approaches to infection control, often surpassing standard care in costs and efficiency.^9^ Several of these interventions have even produced encouraging health gains in a Chilean or regional setting.^5^^,^^13^^,^^14^ However, most previous work has concentrated on unit-based or whole-hospital populations, potentially overlooking heterogeneities in ARB infections and associated risk factors.^7^

Importantly, variations in disease impact and dynamics between sexes have been reported.^15^, ^16^, ^17^, ^18^, ^19^ For example, despite men typically having higher ARB incidence, women generally have a worse prognosis, possibly due to hormonal, behavioural, occupational, and genetic differences that may affect the expression of virulence factors.^17^, ^18^, ^19^, ^20^, ^21^, ^22^ And, while there has been growing recognition of the need for comprehensive sex-based analyses of ARB, this has not yet translated into optimising cost-effective interventions.^15^, ^16^, ^17^

This study aimed to quantify disparities between sexes (defined as biological sex, assigned at birth) regarding CRE and MRSA incidence rates and health outcomes within a Chilean-hospital cohort of infected patients. Then, using a sex-stratified mathematical model for each pathogen, we sought the extent to which leveraging these heterogeneities could improve the cost-effectiveness of more strategically targeted interventions against CRE and MRSA. Our research directly enhances healthcare efficiency by recognising population heterogeneities and addressing disparities in infection burdens.

## Methods

Statistical analyses of patient data collected from three large Chilean hospitals (first stage) informed the parametrisation of a novel compartmental model, adapted from previous literature^23^, ^24^, ^25^ to determine the impact of alternative approaches and targeting strategies to reducing disease burden from MRSA and CRE infections (second stage). The study was approved by the Pontificia Universidad Católica Chile Human Research Ethics Committee (Protocol ID: 200706001).

### First stage: statistical analyses of CRE/MRSA data

Drawing on data from a retrospective matched-parallel cohort study in Chilean tertiary hospitals (2018–2022),^4^^,^^26^ we used descriptive analysis (subgroup means and standard deviations) and inverse probability weighting (IPW) techniques^27^ to analyse the burden of MRSA or CRE in symptomatic hospital patients with positive blood cultures (469 and 404 patients for CRE and MRSA, respectively). We estimated a propensity score to compute IPW via weighting MRSA with methicillin-susceptible *S. aureus* (MSSA) and CRE with carbapenem-susceptible Enterobacterales (CSE) populations according to their baseline characteristics prior to infection (e.g., age, antibiotic consumption, prior hospitalisation, Charlson comorbidity index, community-acquired infection—positive isolate detected within 48 h of hospitalisation—, intensive care unit ‘ICU’ admissions, and biological sex). These characteristics were selected upon expert and literature input, improved Bayesian Information Criterion (BIC), and correlation analysis (using Pearson X^2^) while keeping a Variance Inflation Factor (VIF) < 10. Subsequently, we computed pathogen-specific IPW-adjusted logistic regressions for ICU admission, and survival regressions accounting for competing-risks (i.e., mortality and discharge)^28^ to determine the co-hazards associated with sex, ARB (e.g., MRSA/MSSA or CRE/CSE) and disease severity (e.g., general wards or ICU admission). Marginal effects were computed for sex-specific estimates. Additionally, we collated data on prior antibiotic usage (i.e., methicillin or carbapenems), and length of hospital stays (LOS), across pathogens and patient sex. Further details of the data sources and study population are available in Supplementary Box A1 and elsewhere.^4^

### Second stage: mathematical model

We created a deterministic model, each independently capturing the progression of CRE or MRSA transmission in hospitals through various epidemiological states by adapting existing mathematical models.^29^, ^30^, ^31^ The model directly informs policy by simulating hospital infection dynamics, guiding interventions to manage and prevent CRE and MRSA outbreaks. We included men and women patients (defined as sex assigned at birth) uncolonised (U), colonised but asymptomatic (by either carbapenem/methicillin-resistant ‘C^R^’ or -susceptible ‘C^S^’ Enterobacterales/*S. aureus*), symptomatic with mild (general ward patients) or severe (ICU-admitted patients) infections (I^R^_Mil_ and I^R^_Sev_ for CRE/MRSA and I^S^_Mil_ and I^S^_Sev_ for CSE/MSSA) and dead or recovered (D^R^ or D^S^ and R^R^ or R^S^, respectively, and depending upon antibiotic susceptibility) (Supplementary Figure A1). Mild infections required medical intervention in general wards and severe infections in the ICU. Dual-carriage of susceptible and resistant strains was not incorporated following the assumption that mixed carriage is predominantly sensitive-colonised (CSE or MSSA) and do not further transmit the resistant strain in the absence of antibiotics.^32^ A stable hospital inpatient population was maintained by adjusting daily admissions to daily deaths and discharges. Recovery and treatment were adjusted to the pathogen-specific data. Treatment metrics showed the proportion of patients receiving antibiotics in compliance with pathogen's susceptibility guidelines.^29^ Risk-group specific length of hospital stay, discharge rates, and patient's movement between risk-severity groups within healthcare setting were also included. Individuals could transition between health states daily. See Supplementary material for model description and assumptions, and Supplementary Tables A1 and A2 for full description of the baseline conditions and parameters used to calibrate the model. The ordinary differential equations used to describe system dynamics are in Supplementary Text A1.

### Computational simulations

We computed the transmission parameter by fitting our model to the most recent prevalence rates for CRE/MRSA^14^ utilising the Runge-Kutta^33^ optimisation method. Relative transmission from men and women was informed by the propensity score estimates obtained from the first stage (in the form of odds ratios; OR comparing men relative to women). We did a global sensitivity analysis to identify the parameters that had greatest influence on both CRE/MRSA transmission. Parameter uncertainty was incorporated using the Latin hypercube sampling method with 1000 simulations^34^ and calculated the partial rank correlation coefficient (PRCC).^34^

### Intervention strategies

We evaluated screening (using chromogenic agars and polymerase chain reaction methods ‘PCR’^35^), isolation via enhanced contact precaution (e.g., monitoring and use of gloves and gowns^12^^,^^36^), and decolonisation strategies (mupirocin^37^ and digestive decontamination^38^ for MRSA and CRE, respectively) for each pathogen model (Supplementary Table A3). These strategies were informed by a recent systematic literature review of cost-effective testing-treatment combination strategies.^7^ After computing our base scenario with no intervention (S0), we simulated nine strategies for reducing transmission (S1–S9). S1, S2, and S3 included screening all newly admitted patients using chromogenic agar 48 h, chromogenic agar 24 h, and PCR, respectively, + decolonisation among CRE/MRSA positive. S4, S5 and S6 comprised the same screening diagnostics plus isolation (contact precaution) among CRE/MRSA positives. S7, S8, and S9 involved pre-emptive isolation among all new admissions, and only men or women, respectively. Efficiency parameters for diagnostics, isolation, and treatments are detailed in Supplementary Table A4. We simulated a range of intervention coverage levels and accommodated differential infection risk between sexes by adjusting the success rates of pre-emptive isolation. For example, pre-emptively isolating only men would isolate 1.37-fold more MRSA infectious individuals relative to isolating an equivalent number of patients of both sexes because the OR of MRSA infections among women was 0.73 (equivalent to 1.37 among men, if the reference group was reversed, i.e., 1/0.73).

### Health economics

We evaluated the cost-effectiveness by calculating the incremental cost-effectiveness ratio (ICER) comparing the nine strategies against a ‘do-nothing’ base scenario from a healthcare perspective. We followed the WHO best practices for AMR prevention and control and the Consolidated Health Economic Evaluation Reporting Standards (CHEERs).^39^^,^^40^ We also evaluated the number of averted infected and dead patients associated with CRE/MRSA, and quality-adjusted life years (QALYs) associated with health states. Economic costs included diagnostic, hospital bed-days (general wards, intermediate care, and intensive care units ‘ICU’) and drug costs (i.e., colistin, gentamicin, mupirocin, and chlorhexidine). Hospital bed-days and diagnostic tests' costs were sourced from Chile's public health insurance program,^41^ while antibiotic costs for decolonisation schemes were extracted from Chile's government unit responsible for procuring and distributing drugs and medical supplies.^42^ All costs were expressed in 2022 USDs and no discount rate was applied because we used a one-year time horizon. We reported the ICER per QALY gained for each strategy assessed (Supplementary Tables A4 and A5). Finally, we extrapolated our results to the national level, considering 37,397 hospital beds in the country.^43^ Willingness-to-pay ‘WTP’ was defined following Chile's gross-domestic product (GDP) per capita ($15,356). We calculated the net benefit (‘NB’= (WTP per QALY—cost per QALY)∗QALYs gained) of each intervention, compared to base scenario, and following coverage at 20, 40, 60, 80 and 100% of total country's hospital beds (n = 30,000 beds, excluding those used for psychiatric care).^43^ We provided lower and upper bounds for QALYs gained, and NB were computed adjusting our models to each pathogen's estimated prevalences.^14^

Probability sensitivity analysis (PSA) was performed for intervention parameters using 1000 random samples. We varied diagnostic sensitivities, test turnaround time, isolation and decolonisation efficiency, and health utilities, utilising a beta distribution for rates and gamma distribution for costs and turnaround time. We estimated the percentage of cost-effective (ICER < WTP) simulations per strategy at different WTP thresholds.

All statistical analyses were performed in R version 3.3.4. Full code is available at https://bit.ly/3sgnmrU.

### Role of the funding source

The funder of the study had no role in study design, data collection, data analysis, data interpretation, or writing of the report. Authors had full access to all the data in the study and had final responsibility for the decision to submit for publication.

## Results

### First stage: epidemiology of sex-stratified CRE/MRSA

**Fig. 1:**
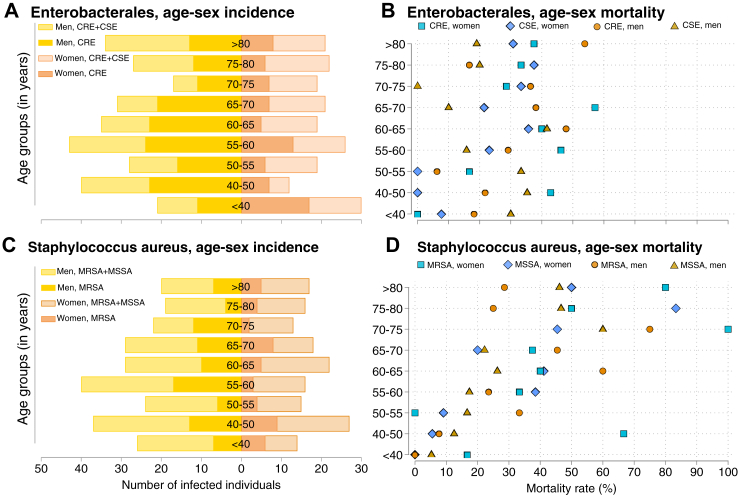
Number of infections and mortality rates among Enterobacterales and *S. aureus* species in three representative hospitals in Chile, 2018–2021. Notes: MRSA, Methicillin-resistant *Staphylococcus aureus*; MSSA, Methicillin-susceptible *Staphylococcus aureus*; CRE, Carbapenem-resistant Enterobacterales; CSE, Carbapenem-susceptible Enterobacterales. Infections were confirmed with blood-cultures. (A) Incidence of Enterobacterales infections by age-sex groups. (B) Average mortality rate among Enterobacterales by age-sex groups. (C) Incidence of Staphylococcus aureus infections by age-sex groups. (D) Average mortality rate among Staphylococcus aureus by age-sex groups.

Supplementary Tables A6 and A7 and Fig. 1 show the sex-stratified incidence and main characteristics of symptomatic infection for Enterobacterales (56.1%, 156/278, with CRE among men, and 40.8%, 78/191, with CRE among women, χ^2^ p < 0.0001) and *S. aureus* (35.2%, 87/247, with MRSA among men, and 29.3%, 46/157, with MRSA among women, χ^2^ p = 0.042). Women diagnosed with MRSA had higher mortality and ICU admissions after diagnosis compared to men (45.6%, 21/46, versus 34.5%, 30/87, χ^2^ p = 0.034; and 45.7%, 21/46, versus 37.9%, 33/87, χ^2^ p = 0.038, respectively). Although the mortality rates were similar, women with CRE had an extended average LOS compared to men (29.9 days versus 24.3 days, respectively; χ^2^ p = 0.049). Our propensity score estimation indicated that women were 0.44 (95% CI = 0.28–0.70, p = 0.0013) and 0.73 (95% CI = 0.48–1.01, p = 0.050) times less likely to have resistant infections among those infected with Enterobacterales and *S. aureus*, respectively (Supplementary Tables A8, A9 and Supplementary Figures A3, A4 for density checks). Our IPW-adjusted models suggested that women's ICU admissions following *S. aureus* infection were generally higher, with a significantly greater ICU admission rate among women with MRSA versus men with MSSA (Supplementary Table A10 and Supplementary Figures A5, A6 for sex-marginal effects). Supplementary Table A11 and Supplementary Figures A7, A8 show that women had the highest mortality rate associated with CRE and MRSA in survival models with competing risks (hazard ratio HR = 2.40, 95% CI = 1.5–3.9, p < 0.0001 and HR = 2.34, 95% CI = 1.3–4.1, p = 0.0031 for women in the ICU if compared to the lowest mortality rate exhibited in men with CSE or MSSA in general wards, respectively).

### Second stage: mathematical model

**Fig. 2:**
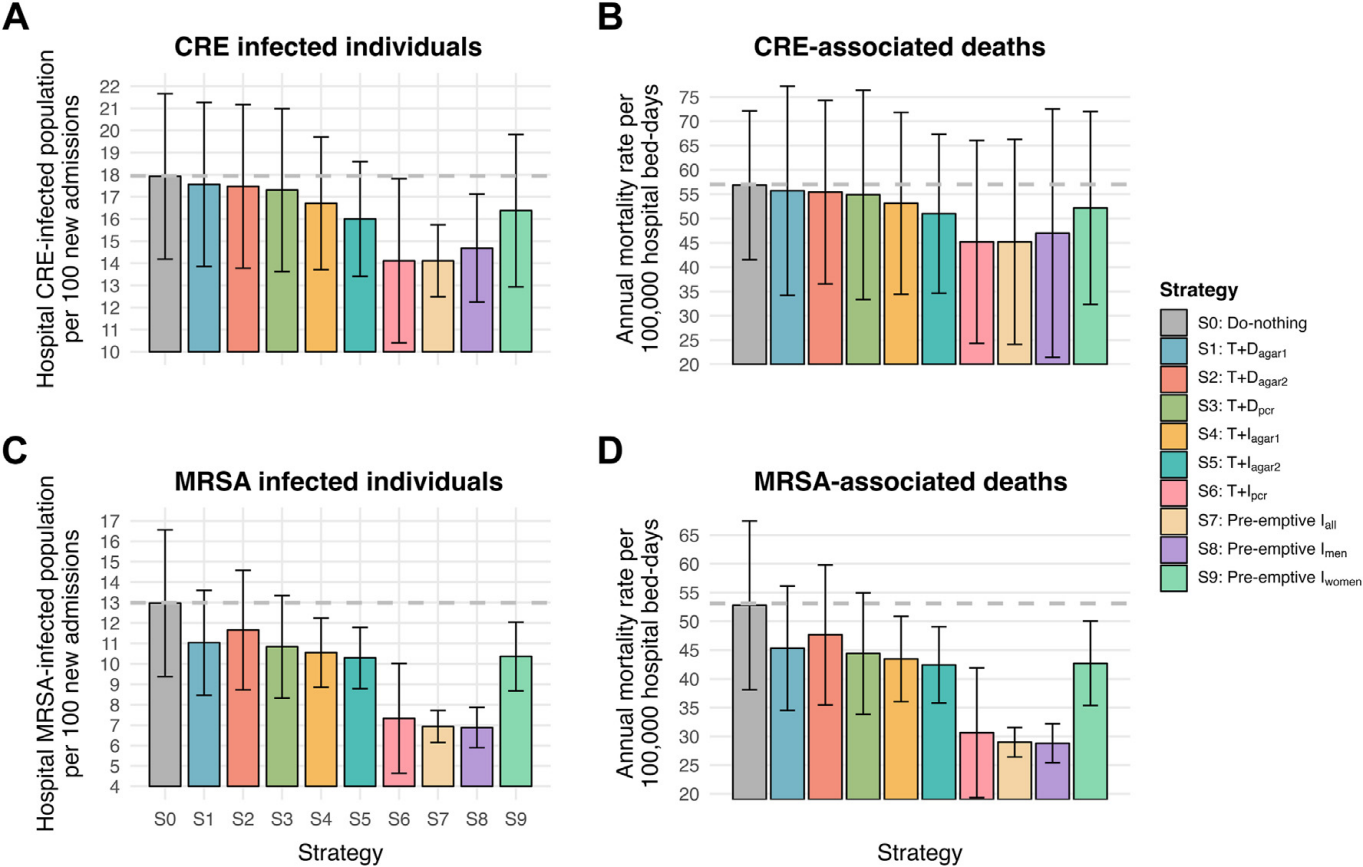
CRE/MRSA impacts in number of infected individuals and number of deaths from the mathematical model, by strategy scheme. Notes: CRE, Carbapenem-resistant Enterobacterales; MRSA, Methicillin-resistant *Staphylococcus aureus*. Dashed line sets the maximum according to S0 strategy for comparison purposes. 95% uncertainty intervals (brackets) were computed utilising pathogen's prevalence upper and lower bounds. T + D = testing + decolonization treatment. T + I= Testing + isolation (contact precaution). Pre-emptive I= Pre-emptive solation (contact precaution). (A) Hospital CRE-infected populations per 100 new admissions. (B) Annual mortality rate per 100,000 hospital bed-days. (C) Hospital MRSA-infected populations per 100 new admissions. (D) Annual mortality rate per 100,000 hospital bed-days.

Pre-emptive isolation and testing plus isolation strategies improved health outcomes and reduced costs the most, regardless of the pathogen (Fig. 2 and Table 1). The best strategy for CRE was pre-emptively isolating men, with an ICER of $1366 per QALY gained (Table 1). The most cost-effective screening strategy for CRE used PCR (ICER = $3156) and chromogenic agar enriched with carbapenems (ICER = $3157), although the former strategy averted double the number of infections and associated deaths. All CRE decolonisation strategies had ICER exceeding the WTP threshold due to their low efficacy. For MRSA, chromogenic agar enriched with salt and oxacillin was preferred for screening carriers, offering better value for money. Testing plus MRSA decolonisation among all new admissions, totalled ICERs of $2099, $1900, and $1850 per QALY gained, respectively, reducing annual deaths per 1000 hospital beds (i.e, [number of deaths/1000 hospital beds]∗365) by 9.9, 3.7, and 5.8, respectively. This strategy was cost-effective and preferred to all test plus isolation interventions because isolated patients continue to contribute to within-hospital transmission (e.g., via healthcare workers) whereas decolonised patients do not. The most cost-effective strategy for MRSA was pre-emptive isolation of newly admitted men (ICER = $1083, saving 32.1 deaths per 1000 hospital beds annually).

**Table 1:** Base-case scenario and cost-effectiveness per strategy over a year's time in a 1000-beds hospital^a^.

Pathogen	Strategy	Population	Scheme	Costs ($)	QALYs (total)	ICER ($/QALY)	Number of infections averted	Number of deaths averted
CRE	Do-nothing	N/A	–	17,857,343 (17.56; 18.1 million)	349,173 (348,380; 350,124)	Ref.	Ref.	Ref.
	Testing + decolonisation	All new admissions	**i)** Chromogenic agar 48 h	18,949,142 (18.66; 19.19 million)	349,232 (348,442; 350,177)	18,586 (17,505; 20,783)	957 (786; 1068)	190 (166; 206)
			**ii)** Chromogenic agar 24 h	19,125,883 (18.84; 19.36 million)	349,247 (348,457; 350,190)	17,270 (16,279; 19,263)	1168 (960; 1306)	237 (207; 257)
			**iii)** PCR	20,152,344 (19.87; 20.39 million)	349,274 (348,486; 350,216)	22,745 (21,515; 25,182)	1499 (1231; 1677)	325 (286; 350)
	Testing + isolation (contact precaution)	All new admissions	**i)** Chromogenic agar 48 h	18,669,352 (18.41; 18.88 million)	349,366 (348,677; 350,206)	4216 (2611; 10,334)	4401 (1801; 7012)	641 (262; 1021)
			**ii)** Chromogenic agar 24 h	18,815,998 (18.58; 19.06 million)	349,477 (348,845; 350,255)	3157 (1942; 7738)	6942 (2877; 10,989)	1011 (419; 1600)
			**iii)** PCR	19,760,327 (19.58; 19.90 million)	349,776 (349,279; 350,396)	3157 (2003; 7396)	13,793 (5959; 21,297)	2008 (867; 3102)
	Pre-emptive isolation (contact precaution)	All and sex-specific new admissions	**i)** All new admissions	19,928,113 (19.75; 20.07 million)	349,776 (349,279; 350,396)	3435 (2189; 8014)	13,792 (5959; 21,297)	2008 (867; 3102)
			**ii)** Men newly admitted	18,743,732 (18.55; 18.92 million)	349,822 (349,232; 350,451)	1366 (960; 3029)	11,680 (5629; 16,232)	1700 (819; 2364)
			**iii)** Women newly admitted	18,924,151 (18.69; 19.15 million)	349,525 (348,784; 350,317)	3034 (2586; 5812)	5563 (2983; 6559)	809 (434; 955)
MRSA	Do-nothing	N/A	–	17,718,562 (17.38; 17.94 million)	347,290 (346,165; 349,123)	Ref.	Ref.	Ref.
	Testing + decolonisation	All new admissions	**i)** Chromogenic agar 48 h	18,224,547 (17.93; 18.32 million)	347,531 (346,676; 349,256)	2099 (746; 4154)	6699 (3060; 17,206)	996 (476; 2401)
			**ii)** Chromogenic agar 24 h	18,445,419 (18.13; 18.61 million)	347,459 (346,474; 349,223)	4287 (2156; 7603)	4442 (2079; 9807)	685 (347; 1416)
			**iii)** PCR	19,483,761 (19.18; 19.59 million)	347,567 (346,759; 349,285)	6357 (2779; 11,145)	7153 (3267; 19,544)	1118 (562; 2756)
	Testing + isolation (contact precaution)	All new admissions	**i)** Chromogenic agar 48 h	18,439,084 (18.18; 18.49 million)	347,588 (346,850; 349,198)	2411 (806; 10,859)	8643 (1850; 22,860)	1248 (269; 3250)
			**ii)** Chromogenic agar 24 h	18,616,308 (18.37; 18.66 million)	347,621 (346,917; 349,207)	2709 (956; 11,874)	9590 (2077; 25,175)	1384 (302; 3581)
			**iii)** PCR	19,576,302 (19.39; 19.62 million)	347,993 (347,381; 349,337)	2641 (1379; 9447)	20,495 (5307; 40,933)	2960 (772; 5886)
	Pre-emptive isolation (contact precaution)	All and sex-specific new admissions	**i)** All new admissions	19,801,550 (19.62; 19.86 million)	348,044 (347,413; 349,361)	2760 (1538; 9454)	22,014 (5901; 41,977)	3180 (858; 6042)
			**ii)** Men newly admitted	18,542,668 (18.41; 18.42 million)	348,050 (347,725; 349,338)	1083 (307; 4828)	22,209 (5330; 53,858)	3206 (775; 7682)
			**iii)** Women newly admitted	18,802,155 (18.54; 18.93 million)	347,630 (346,726; 349,228)	3185 (1773; 11,198)	9302 (2462; 17,304)	1349 (359; 2512)

Notes: CRE, Carbapenem-resistant Enterobacterales; ICER, Incremental cost-effectiveness ratio; MRSA, Methicillin-resistant *Staphylococcus aureus*; N/A, Not applicable; PCR, polymerase chain reaction; QALYs, Quality-adjusted life years; Ref., Reference.

a Model estimations specify upper and lower bounds within brackets (uncertainty intervals), calibrated to the national prevalence extremes of the pathogen. QALYs are reported as totals per intervention type.

PCR testing combined with isolation strategies led to a 22.2% and 30.8% decrease in CRE infections per 100 admissions and deaths per 100,000 hospital-bed days, respectively, compared to a do-nothing scenario (Fig. 2, panel A and B). Similarly, MRSA infections decreased by 46.2% and deaths by 41.5% (Fig. 2, panel C and D). Isolating men pre-emptively led to similar figures. The health benefits per new admission were greatest when pre-emptively isolating men, with 0.0121 and 0.014 incremental QALYs and $14.9 and $16.8 incremental costs per new admission among CRE and MRSA, respectively, compared to a do-nothing scenario (Supplementary Figure A9).

### Probability sensitivity analysis (PSA) and willingness-to-pay thresholds

Supplementary Figures A10 and A11 present the PSA analysis per pathogen and intervention strategy, suggesting consistent increased effectiveness in 100% scenarios and positive average incremental costs. Utilising PSA results, the ICER fell below the WTP thresholds of $8000 and $16,000 per QALY gained in 80% and 100% of simulations, respectively, for pre-emptive isolation strategies (covering all new admissions and high-risk men) and the PCR plus isolation approach in CRE/MRSA models (Supplementary Figure A12). Agar-based screening combined with isolation was deemed cost-effective in over 81% of simulations at WTP = $15,356 per QALY. Notably, for MRSA, strategies involving 48-h agar screening and PCR combined with decolonisation were cost-effective in more than 80% of simulations, using the national WTP threshold. However, decolonisation schemes for CRE only reached cost-effectiveness in 55% of simulations at the country's WTP threshold.

### Analysis of the intervention strategies impact at national scale

The most favourable cost-effectiveness outcomes were observed following pre-emptive isolation of high-risk individuals, specifically men, or all new admissions, as well as PCR screening of new admissions followed by isolation of those testing positive for CRE/MRSA (Table 2). Implementing these strategies in at least 20% of national hospital beds could yield monetary benefits exceeding $47.4 millions per year. Increasing this coverage to 40% shows the most substantial incremental gain more than doubling monetary savings, while extending to 80% coverage produces net benefits ranging between $189.7 and $276.8 million, depending on the strategy. Conversely, widespread adoption of digestive decolonisation approaches for CRE could result in financial deficits across all levels of hospital-bed coverage, as indicated by a negative NB.

**Table 2:** National scale cost savings and potential health benefits among selected strategies.

Pathogen	Strategy	Scheme	National coverage (total hospital beds, %)	QALYs gained	95% UI_QALYs_	NB ($)	95% UI_NB_ (in 1000s $)
CRE	Testing + decolonisation, all new admissions	**i)** Chromogenic agar 48 h	20	354	318; 372	−821,634	−1437; −461
			40	708	636; 744	−1,643,268	−2873; −923
			60	1062	954; 1116	−2,464,902	−4310; −1384
			80	1416	1272; 1488	−3,286,536	−5747; −1845
			100	1770	1590; 1860	−4,108,170	−7184; −2306
		**ii)** Chromogenic agar 24 h	20	444	396; 462	−446,220	−1187; −6
			40	888	792; 924	−892,440	−2374; −13
			60	1332	1188; 1386	−1,338,660	−3562; −19
			80	1776	1584; 1848	−1,784,880	−4749; −26
			100	2220	1980; 2310	−2,231,100	−5936; −32
		**iii)** PCR	20	606	552; 636	−3,926,880	−4922; −3339
			40	1212	1104; 1272	−7,853,760	−9844; −6678
			60	1818	1656; 1908	−11,780,640	−14,767; −10,017
			80	2424	2208; 2544	−15,707,520	−19,689; −13,356
			100	3030	2760; 3180	−19,634,400	−24,611; −16,695
	Testing + isolation (contact precaution), all new admissions	**i)** Chromogenic agar 48 h	20	1158	492; 1782	13,952,742	2918; 24,331
			40	2316	984; 3564	27,905,484	5836; 48,663
			60	3474	1476; 5346	41,858,226	8754; 72,994
			80	4632	1968; 7128	55,810,968	11,672; 97,326
			100	5790	2460; 8910	69,763,710	14,590; 121,657
		**ii)** Chromogenic agar 24 h	20	1824	786; 2790	23,908,992	6702; 39,961
			40	3648	1572; 5580	47,817,984	13,404; 79,922
			60	5472	2358; 8370	71,726,976	20,107; 119,884
			80	7296	3144; 11,160	95,635,968	26,809; 159,845
			100	9120	3930; 13,950	119,544,960	33,511; 199,806
		**iii)** PCR	20	3618	1632; 5394	47,424,744	14,474; 76,929
			40	7236	3264; 10,788	94,849,488	28,948; 153,858
			60	10,854	4896; 16,182	142,274,232	43,423; 230,788
			80	14,472	6528; 21,576	189,698,976	57,897; 307,717
			100	18,090	8160; 26,970	237,123,720	72,371; 384,646
	Pre-emptive isolation (contact precaution), all new admissions and sex-specific	**i)** All new admissions	20	3618	1632; 5394	46,418,940	13,466; 75,926
			40	7236	3264; 10,788	92,837,880	26,931; 151,852
			60	10,854	4896; 16,182	139,256,820	40,397; 227,778
			80	14,472	6528; 21,576	185,675,760	53,863; 303,704
			100	18,090	8160; 26,970	232,094,700	67,328; 379,630
		**ii)** Men newly admitted	20	3894	1962; 5112	58,016,706	25,969; 78,239
			40	7788	3924; 10,224	116,033,412	51,938; 156,478
			60	11,682	5886; 15,336	174,050,118	77,907; 234,717
			80	15,576	7848; 20,448	232,066,824	103,876; 312,957
			100	19,470	9810; 25,560	290,083,530	129,845; 391,196
		**iii)** Women newly admitted	20	2112	1158; 2424	27,943,872	12,105; 33,158
			40	4224	2316; 4848	55,887,744	24,209; 66,316
			60	6336	3474; 7272	83,831,616	36,314; 99,474
			80	8448	4632; 9696	111,775,488	48,418; 132,632
			100	10,560	5790; 12,120	139,719,360	60,523; 165,789
MRSA	Testing + decolonisation, all new admissions	**i)** Chromogenic agar 48 h	20	1446	798; 3066	20,484,036	9665; 47,581
			40	2892	1596; 6132	40,968,072	19,329; 95,163
			60	4338	2394; 9198	61,452,108	28,994; 142,744
			80	5784	3192; 12,264	81,936,144	38,658; 190,325
			100	7230	3990; 15,330	102,420,180	48,323; 237,906
		**ii)** Chromogenic agar 24 h	20	1014	600; 1854	12,145,692	5197; 26,158
			40	2028	1200; 3708	24,291,384	10,394; 52,316
			60	3042	1800; 5562	36,437,076	15,592; 78,474
			80	4056	2400; 7416	48,582,768	20,789; 104,632
			100	5070	3000; 9270	60,728,460	25,986; 130,790
		**iii)** PCR	20	1662	972; 3564	16,467,096	4977; 48,064
			40	3324	1944; 7128	32,934,192	9953; 96,128
			60	4986	2916; 10,692	49,401,288	14,930; 144,192
			80	6648	3888; 14,256	65,868,384	19,907; 192,256
			100	8310	4860; 17,820	82,335,480	24,883; 24,0321
	Testing + isolation (contact precaution), all new admissions	**i)** Chromogenic agar 48 h	20	1788	450; 4110	24,770,952	2433; 63,536
			40	3576	900; 8220	49,541,904	4865; 127,073
			60	5364	1350; 12,330	74,312,856	7298; 190,609
			80	7152	1800; 16,440	99,083,808	9731; 254,146
			100	8940	2250; 20,550	123,854,760	12,164; 317,682
		**ii)** Chromogenic agar 24 h	20	1986	504; 4512	26,922,216	2213; 69,074
			40	3972	1008; 9024	53,844,432	4426; 138,148
			60	5958	1512; 13,536	80,766,648	6639; 207,223
			80	7944	2016; 18,048	107,688,864	8852; 276,297
			100	9930	2520; 22,560	134,611,080	11,065; 345,371
		**iii)** PCR	20	4218	1284; 7296	57,466,032	8754; 108,608
			40	8436	2568; 14,592	114,932,064	17,509; 217,217
			60	12,654	3852; 21,888	172,398,096	26,263; 325,825
			80	16,872	5136; 29,184	229,864,128	35,017; 434,433
			100	21,090	6420; 3,6480	287,330,160	43,772; 543,041
	Pre-emptive Isolation (contact precaution), all new admissions and sex-specific	**i)** All new admissions	20	4524	1428; 7488	61,096,620	9726; 110,276
			40	9048	2856; 1,4976	122,193,240	19,452; 220,552
			60	13,572	4284; 22,464	183,289,860	29,178; 330,827
			80	18,096	5712; 29,952	244,386,480	38,904; 441,103
			100	22,620	7140; 37,440	305,483,100	48,631; 551,379
		**ii)** Men newly admitted	20	4560	1290; 9360	69,229,920	14,754; 149,367
			40	9120	2580; 18,720	138,459,840	29,507; 298,734
			60	13,680	3870; 28,080	207,689,760	44,261; 448,101
			80	18,240	5160; 37,440	276,919,680	59,015; 597,468
			100	22,800	6450; 46,800	346,149,600	73,769; 746,834
		**iii)** Women newly admitted	20	2040	630; 3366	26,683,200	3192; 48,780
			40	4080	1260; 6732	53,366,400	6384; 97,560
			60	6120	1890; 10,098	80,049,600	9577; 146,340
			80	8160	2520; 13,464	106,732,800	12,769; 195,120
			100	10,200	3150; 16,830	133,416,000	15,961; 243,900

Notes: CRE, Carbapenem-resistant Enterobacterales; MRSA, Methicillin-resistant *Staphylococcus aureus*; NB, Net benefit; PCR, polymerase chain reaction; QALYs, Quality-adjusted life years; UI, Uncertainty intervals; WTP, Willingness to Pay. 95% UIs were estimated using pathogen-specific prevalence and their upper and lower bounds. NB is calculated as (WTP per QALY—cost per QALY) ∗(QALYs gained compared to S0).

### Global sensitivity analysis of the model

Supplementary Figure A13 shows the results from the global sensitivity analysis. For the CRE model, key factors included the transmission parameter (PRCC = 0.37, p < 0.0001), discharge rates for uncolonized individuals (men: PRCC = 0.38, women: PRCC = 0.31, p < 0.0001 and p = 0.007, respectively), and rates of CRE clearance and treatment (PRCC = −0.52 and −0.38, p < 0.0001). In the MRSA model, significant parameters were the clearance rate and treatment rate among men (PRCC = −0.47 and −0.32, p < 0.0001), while transmission rate, discharge rate among women, and progression to infection from MRSA colonisation had significant positive influence on MRSA burden (PRCC = 0.58, 0.35, 0.31; p < 0.0001, p = 0.0012, and p = 0.0043, respectively). Sensitivity of diagnostic tests and result delays were also analysed, showing that a 10% increase in test sensitivity could prevent from 72 to 164 CRE and 125 to 183 MRSA infections (Supplementary Figures A14 and A15), if test turnaround was kept constant at three or one day, respectively. Reducing turnaround from three days to one day could prevent up to 1242 CRE and 1713 MRSA infections, assuming constant test sensitivity.

## Discussion

Analysing CRE and MRSA rates and outcomes in Chilean tertiary care hospitals revealed distinct epidemiological patterns between patients of different sex. We used mathematical modelling to test a combination of strategies to reduce the burden of CRE/MRSA in hospitals, capitalising on these patterns to optimise intervention cost-effectiveness.

Our results showed significant sex-based differences in both incidence and clinical outcomes. Men had higher CRE/MRSA incidence rates corroborating previous findings both in the region^44^ and elsewhere.^17^^,^^45^ The reasons for these differences are uncertain but could be due to sex-specific risk factors that predispose men to CRE/MRSA acquisition (e.g., diabetes, indwelling devices).^15^^,^^17^ Our data suggested that observed sex differences were associated with elevated antibiotic consumption, increased exposure to risk factors (e.g., indwelling catheters, primary infection sources), and higher rates of kidney therapy and mechanical ventilation prior to infection diagnosis among men. Contrarily, after accounting for major confounders, we found that mortality was higher among women, experiencing more severe disease prognosis. For instance, we found a significantly longer LOS before infection diagnosis, higher ICU admissions and increased needs for mechanical ventilation and post-infection surgery. This result is also corroborated by previous studies,^46^^,^^47^ including a meta-analysis that showed a 1.18-fold increase in *S. aureus* severity and mortality among women.^48^ Higher mortality has also been shown among women with hospital-acquired bloodstream infections^47^ and severe sepsis.^49^ Potential reasons include behavioural factors like delayed care-seeking, treatment postponements, and lower quality of acute care compared to men.^50^

As is the case for most countries,^51^ Chile's national action plan does not accommodate these differences.^5^ Even where national action plans do explicitly highlight this characteristic, such as Denmark's plan acknowledging potential differences in antibiotic usage and infection burdens, these have not translated into sex-specific intervention targeting.^5^ To our knowledge, no country explicitly includes this as a consideration in their national action plan. Our study's results advocate for accounting for sex differences when devising control strategies. Pre-emptive isolation or contact precaution that targeted men yielded the most beneficial returns (lowest ICER). We found that isolating high-risk men carriers of CRE/MRSA indirectly protected women by curbing pathogen spread in healthcare environments, which is potentially due to less exposure in risk factors among this subgroup (e.g., indwelling devices, antibiotic consumption). PCR testing coupled with contact precaution demonstrated best value for money among CRE and MRSA individuals. We observed lower ICERs, compared to UK- and US-based studies ($13,904/life year saved^52^ and $80,159/QALY^9^ gained for MRSA), because of the increased costs associated with bed-days and low MRSA prevalence in those country settings (≈2%).^9^^,^^52^ Several novel diagnostic tools are under current development for CRE and/or MRSA (e.g., Multiplex nucleic amplification tests), and our analysis of the trade-offs between diagnostic delays and test-sensitivity supports development of their target product profiles.

Consistent with our results, the use of mupirocin for MRSA has been shown to be a cost-effective strategy when coupled with PCR screening.^53^ Reported ICERs have ranged from dominance (ICER <0) to $11,005/QALY gained.^9^^,^^54^, ^55^, ^56^ While one study suggested an association between decolonisation with nasal mupirocin and a rise in infections by alternative microorganisms, the causal relationship was unknown.^37^ Decolonisation strategies for CRE were the least cost-effective in our analysis. We modelled gentamicin and colistin selective digestive decontamination for CRE decolonisation treatment, but evidence is limited and there is no consensus over its effectiveness or safety.^57^^,^^58^ Our modelled CRE decolonisation failed to meet ICER < country WTP, aligning with recent European Committee on Infection Control guidelines which recommended against its usage.^59^ Several alternative interventions have emerged in recent years, including faecal microbiota transplantation, use of probiotics and bacteriophages, although many of these will likely be costlier than contact precaution strategies.^60^^,^^61^ Although contact-precaution measures should be taken with caution as could have no effective impact in wards with extensive surveillance screening, as shown in Europe.^62^ A promising development is the arrival of new CRE treatments, such as ceftazidime/avibactam which was recently shown to halve 30-day mortality compared to colistin-based regimen.^63^ This could prove particularly valuable in Chile where carbapenemase-producing *Klebsiella pneumoniae* was reported to increase from 12.8% pre–COVID-19 to 51.9% after the pandemic.^64^ However, caution in antibiotic use is crucial, as preventive measures are essential to preserve antibiotic effectiveness, protect the microbiome, and reduce opportunistic infections and related disease burdens.^65^^,^^66^ Narrow-spectrum antibiotics can further reduce microbiome disruption, resistance development, and complications like *Clostridioides difficile* infections.

Our study has some limitations in terms of the sampling of patients and the assumptions made with the analysis and mathematical model. Patient data from three tertiary care hospitals were selected for their representativeness of the broader population. Despite efforts to control for key confounders in the target symptomatic populations, it is possible that some confounders were overlooked (e.g., time-varying characteristics after infection onset, prior pathogen colonisation), potentially biasing the results (i.e., unmeasured confounding among sex-specific characteristics). However, our study results and patient characteristics were consistent with a prior study only exploring colonisation.^67^ As with all mathematical models, ours only represents a highly simplified structure underlying what were perceived to be the key processes involved in CRE/MRSA transmission and health outcomes. For example, we did not include co-infections (though, these are likely rare), nor did we account for within-host dynamics and interactions between resistant and susceptible strains of pathogens. Once these processes are better understood, the model can easily be adapted to account for this additional complexity. Further, despite the relative simplicity of the model, its parametrisation necessitated gleaning estimates from studies conducted in different locations/populations with their own epidemiology. Again, this is a common shortcoming of models but by conducting a comprehensive sensitivity analysis, we have identified the most influential parameters and quantified their impacts in our outcomes of interest. We excluded chlorhexidine bathing due to mixed evidence on effectiveness^68^ and because it is currently integrated into some existing local budgets and practices.^69^ Moreover, we did not apply hospital-specific dynamics, as data on CRE/MRSA incidence over time were unavailable at the hospital level, despite potential variability across hospitals. Data on gender and ethnicity were unavailable, limiting demographic analysis. Finally, we did not account for the challenges of implementing high-quality isolation, nor the mental health burden on patients or potential declines in care quality, potentially underestimating the full impact of these interventions.

Our study underscores the importance of identifying key risk factors associated with resistant infections and then utilising this information to strategize interventions and improve patient health outcomes. Statistical analysis of Chilean hospital patient data identified that men were likely the key source of CRE/MRSA transmission while women typically suffered worse health outcomes (i.e., prognosis). Our model demonstrated that capitalising on this epidemiological understanding by pre-emptively reducing transmission from men could yield considerable health gains. Alternative definitions of monetary intervention acceptability, such as the WHO's cost-effectiveness threshold of three-times the GDP per capita, suggested that our tested interventions could be highly cost-effective. The transferability of this strategy to other settings and pathogens warrants further consideration. Additionally, exploring sex-specific risk factors for ARB acquisition may inform cost-effective interventions to reduce the burden of resistant infections.

## Contributors

Conceptualization, KA, LY; methodology, KA, LY; data collation and extraction, KA; Chilean cohort data: PG, JM, EU; Funding acquisition: KA, PG, JM, EU; formal analysis, KA; writing—original draft preparation, KA; writing—review and editing, KA, PG, AP, JM, EU, LY; Project administration: AP, JM, EU; supervision, LY. All authors have read and approved the final version of the manuscript.

## Data sharing statement

Data are available in previous literature and full code used for analyses is available in Github at www.bit.ly/3sgnmrU.

## Declaration of interests

The authors declare no competing interest.

## Appendix A Supplementary data

Abstract_spanish

ModellingChile
