# Supplementary material for: Cost-effectiveness of screening, decolonisation and isolation strategies for carbapenem-resistant Enterobacterales and methicillin-resistant Staphylococcus aureus infections in hospitals: a sex-stratified mathematical modelling study
Source: Lancet Reg Health Am. 2025 Feb 15;43:101019. doi: 10.1016/j.lana.2025.101019 (PMC11872075; doi:10.1016/j.lana.2025.101019)
Supplement: Abstract_spanish [file mmc1.docx]

*This translation in Spanish was submitted by the authors and we reproduce it as supplied. It has not been peer reviewed. Our editorial processes have only been applied to the original abstract in English, which should serve as reference for this manuscript.*

**Resumen**

**Antecedentes**. El *Staphylococcus aureus* resistente a meticilina (MRSA) y los enterobacterales resistentes a carbapenémicos (CRE) imponen la mayor carga entre los patógenos bacterianos críticos. La evidencia sobre las diferencias de sexo en las infecciones bacterianas resistentes a los antibióticos está aumentando, pero es necesario enfocarse en las implicaciones para las políticas.
**Métodos**. Evaluamos el impacto de CRE/MRSA en la estancia hospitalaria prolongada, la admisión a la unidad de cuidados intensivos y la mortalidad por sexo en un estudio de cohorte retrospectivo (n=873) de pacientes en tres hospitales chilenos, 2018-2021. Utilizamos puntuaciones de propensión y ponderación por probabilidad inversa combinadas con análisis descriptivos, logísticos y de riesgos competitivos. Desarrollamos un modelo compartimental determinístico estratificado por sexo para analizar las dinámicas de transmisión hospitalaria y la rentabilidad de nueve intervenciones. Comparamos las intervenciones según la razón de costo-efectividad incremental (ICER) por año de vida ajustado por calidad (QALY) ganado y estimamos los beneficios netos.
**Resultados**. Las probabilidades ajustadas de que las mujeres adquirieran CRE y MRSA fueron de 0,44 (0,28-0,70; p=0,001) y 0,73 (IC 95%= 0,48-1,01; p=0,050), respectivamente. Los modelos de riesgos competitivos indicaron tasas de mortalidad más altas entre las mujeres en comparación con los hombres. Las proyecciones del modelo matemático mostraron que el aislamiento preventivo de todos los hombres de alto riesgo recién admitidos fue la intervención más costo-efectiva (ICER=$1366/QALY y $1083/QALY para CRE y MRSA, respectivamente). El agar cromogénico junto con la descolonización de MRSA fue la segunda intervención más costo-efectiva ($2099/QALY), seguida de estrategias de cribado más aislamiento o aislamiento preventivo (ICER entre $2411/QALY y $4216/QALY para los modelos de CRE y MRSA). El análisis de sensibilidad probabilístico mostró que las estrategias eran ICER<disposición a pagar en el 80% de las simulaciones, excepto para la prueba más descolonización digestiva para CRE. Con una cobertura hospitalaria nacional del 20%, se podrían ahorrar al menos $12,2 millones.
**Interpretación**. Nuestro modelo sugiere que las estrategias de control de infecciones dirigidas abordarían eficazmente el aumento de las infecciones por CRE y MRSA. Maximizar las ganancias en términos de salud y economía puede lograrse enfocándose en medidas de control para los hombres como principales impulsores de la transmisión, reduciendo así la carga desproporcionada de enfermedad que soportan las mujeres.

**Palabras clave**: Modelado matemático, Resistencia a los antibióticos, Dinámica de transmisión, Intervenciones, Costo-efectividad.
