# Supplementary material for: Cost-effectiveness of screening, decolonisation and isolation strategies for carbapenem-resistant Enterobacterales and methicillin-resistant Staphylococcus aureus infections in hospitals: a sex-stratified mathematical modelling study
Source: Lancet Reg Health Am. 2025 Feb 15;43:101019. doi: 10.1016/j.lana.2025.101019 (PMC11872075; doi:10.1016/j.lana.2025.101019)
Supplement: ModellingChile [file mmc2.docx]

**Supplementary Appendix**

**Cost-effectiveness of Screening, Decolonisation and Isolation Strategies for Carbapenem-resistant Enterobacterales and Methicillin-resistant *Staphylococcus aureus* Infections in hospitals: A Sex-stratified Mathematical Modelling Study**

Kasim Allel, Patricia Garcia, Anne Peters, Jose Munita, Eduardo A. Undurraga, Laith Yakob

Contents

[Abbreviations 2](#_Toc179741738)

[I. Epidemiological study 3](#_Toc179741739)

[II. Mathematical model parameters and definitions 4](#_Toc179741740)

[III. Intervention details and economic costs 14](#_Toc179741741)

[IV. Descriptive statistics among populations of interest 18](#_Toc179741742)

[V. Supplementary results: propensity score and inverse probability weights 23](#_Toc179741743)

[VI. Supplementary methods: regression and survival analyses 26](#_Toc179741744)

[VII. Supplementary results: Inverse-probability weighted regression results 26](#_Toc179741745)

[VIII. Mathematical model results 32](#_Toc179741746)

[IX. Guidelines for economic evaluations and transmission models 39](#_Toc179741747)

[X. STROBE guidelines for observational studies 41](#_Toc179741748)

[XI. Additional references 43](#_Toc179741749)

# Abbreviations

| ABR | Antibiotic resistance/resistant |
| --- | --- |
| ABS | Antibiotic susceptible |
| AMR | Antimicrobial resistance/resistant |
| AMS | Antimicrobial susceptible |
| ATB | Antibiotic |
| BIC | Bayesian Information Criterion |
| BSI | Bloodstream infections |
| CDC | Centre for Disease Control |
| CI | Confidence interval |
| CRE | Carbapenem-resistant Enterobacterales |
| CSE | Carbapenem-sensitive Enterobacterales |
| DALYs | Disability-adjusted life years |
| eCDC | European Centre for Disease Control and Prevention |
| ESBL | Extended spectrum beta-lactamase |
| GDP | Gross domestic product |
| GLASS | Global Antimicrobial Resistance and Surveillance System |
| GNI | Gross National Income |
| GW | General wards |
| HICs | High income countries |
| HR | Hazard ratio |
| ICER | Incremental cost-effectiveness ratio |
| ICU | Intensive Care Units |
| IPW | Inverse probability weighting |
| LOS | Length of Stay |
| LMICs | Low- and middle-income countries |
| MDR | Multi-drug resistance |
| MRSA | Methicillin resistant Staphylococcus aureus |
| MSSA | Methicillin susceptible Staphylococcus aureus |
| NB | Net benefit |
| OECD | Organization for Economic Cooperation and Development |
| OR | Odds Ratio |
| PSA | Probability sensitivity analysis |
| QALYs | Quality-adjusted life years |
| SDG | Sustainable Development Goals |
| UN | United Nations |
| VIF | Variance inflation factor |
| WB | World Bank |
| WHO | World Health Organization |
| WTP | Willingness-to-pay |

# I. Epidemiological study

**Box A1.** Main variables collected in the retrospective study

**Epidemiological data from the parallel matched retrospective study.^1^**

The study was carried out during 2018-2021 among hospital inpatients from three of the largest hospitals in Chile (representative of the Chilean population; one in the South, North and Centre of the country). The questionnaire included patient sociodemographic characteristics, information on the pathogen causing bloodstream infection (with special attention drawn to WHO priority list for pathogens) and corresponding cultures taken (blood), hospitalisation-related data, and health status reported. Most variables were collated upon BSI diagnosis (moment when first blood culture was taken).

**I.** Sociodemographic characteristics: Date of birth, age, and sex at birth.

**II.** Pathogen and culture: antimicrobial susceptibility testing, antibiotic family and specific antibiotic tested, name of the pathogen causing the infection, number of blood cultures taken (results [+ or -] and date), use of external devices (central line catheter with date and length of use), location of the patient within the hospital, endocarditis, surgery taken (type and date), recurrent bacteraemia or new (with dates and length, date of clearance was collected), respiratory diagnostic including the presence of symptoms and previous respiratory diseases, symptoms experienced during the hospital stay and after the discharge, secondary source of infection including urinary and wounds and use of urinary catheters with dates and length (if any urinary infection presented).

**III.** *Hospitalisation-related*: previous surgery undertaken (type and date of entry and discharge), number of medical consultations (and dates), antibiotic used in previous hospitalisations (date detailed; and up until 60 days before BSI diagnosis), data of entry/exit to the hospital (reason why they attended the hospital, discharge, and outcome), place of origin of the infection (another hospital, home, homecare, etc), place where the patients were hospitalised (ICU, etc). Length of hospital stay prior to the BSI diagnosis was captured up until 60 days before BSI diagnosis.

**IV.** Comorbidities and health data: CHARLSON score, liver diseases, HIV, kidney therapy, dialysis, transplant, cancer, allergies, cardiac device, pregnancy, intake of any medicine. Also, mechanical ventilation (date and length), antibiotic therapies undergone (with date and duration for each specific antibiotic and frequency), date of death, therapies received (dates and treatments related). Finally, risk factors to assess and evaluate patient’s comorbidities including diabetes, dementia, hepatitis, stroke, heart disease, coronary artery disease, peripheral vascular disease, cerebrovascular disease, severe chronic obstructive pulmonary disease (COPD), connective tissue disease, peptic ulcer disease, liver disease, renal disease, and HIV infection. All these variables were captured at the moment of BSI diagnosis.

**Burden measures**

*Health Burden*: ICU admission and Mortality status are reported (including the registry of the day of death since hospitalisation). Length of hospital stay in days since hospitalisation until discharge/death is also obtained.

*Economic burden*: Hospitalisation costs would be provided including cost per day of stay, equipment used, days of hospitalization, and resources consumed in hospital (number of diagnostic tests and medications). Costs per bed-day by specialty would be also included (medicine, surgery, paediatrics, etc.), together with the costs of patient isolation, staff involved in patient’s treatment (number of hours spent by nurses, medics, physicians, infection, and lab control staff), antimicrobial administration costs, and laboratory costs for screening procedures.

# II. Mathematical model parameters and definitions

*Asymptomatic*

**C^S^**

**D^S^**

**R^S^**

**I^S^_MIL_**

*Symptomatic*

**I^S^_SEV_**

**Hospital population dynamics**

**I^R^_SEV_**

**C^R^**

*Symptomatic*

*Asymptomatic*

**I^R^_MIL_**

**D^R^**

**R^R^**

**U**

Uncolonised with ARB or ASB strain

Asymptomatic, colonised with ARB strain

Symptomatic, Mild infection with ARB strain

Symptomatic, Severe infection with ARB strain

Deceased, associated with ARB strain infections

Recovered/discharged after treatment for ARB infection

**U**

**C^R^**

**I^R^_SEV_**

**I^R^_MIL_**

**D^R^
R^R^**

**C^S^**

**I^S^_SEV_**

**I^S^_MIL_**

**D^S^
R^S^**

Asymptomatic, colonised with ASB strain

Symptomatic, Mild infection with ASB strain

Symptomatic, Severe infection with ASB strain

Deceased, associated with ASB strain infections

Recovered/discharged after treatment for ASB infection

**Figure A1.** Model scheme of in-hospital transmission dynamics of MRSA/CRE. Hospital population dynamics for methicillin-resistant *Staphylococcus aureus* (MRSA) and carbapenem-resistant Enterobacterales (CRE), separately modelled. ARB=Antibiotic resistant bacteria; ASB= Antibiotic-susceptible bacteria. New admissions offset discharges entering to U, C^s^, C^R^, I^S^ and I^R^ compartments

**Model description in full**

Our model delineates the sex-stratified progression and outcomes of individuals within a healthcare setting, segmented by their interaction with Antibiotic-Resistant Bacteria (ARB) and Antibiotic-Sensitive Bacteria (ASB) strains. For this study purposes we referred about MRSA/MSSA and CRE/CSE if talking about ARB/ASB. Our model was structured into distinct compartments:

Uncolonized: Individuals not carrying ARB or ASB strains, susceptible to colonisation.

Asymptomatic, Colonised: Individuals silently carrying ARB or ASB strains without displaying symptoms.

Symptomatic, Mild Infection: Individuals showing mild symptoms due to infection attributable to ARB or ASB strains, requiring minimal medical intervention in a general ward.

Symptomatic, Severe Infection: Patients manifesting severe symptoms from infections caused by ARB or ASB strains, necessitating intensive care within hospital’s ICUs.

Deceased: Patients who succumb to infections caused by ARB or ASB strains.

Recovered/Discharged: Individuals who have recovered from ARB or ASB infections and have been discharged following treatment.

The dynamics involve transitions from being uncolonised to becoming asymptomatically colonized upon exposure, defined by a force-of-infection. Colonised individuals may progress to mild or severe symptomatic states based on strain virulence (CRE/MRSA) and host factors (sex-based differential progressions based on data from the retrospective study). Mild and severe infections may lead to mortality (parametrised accordingly and following sex-specific distributions from the cohort study), while successful treatment (derived using cephalosporins or vancomycin for CRE and MRSA, respectively, and length-of hospital stays from the cohort study) results in recovery and discharge. The model captures different infection trajectories for ARB and ASB strains, reflecting variations in treatment efficacy, infection control practices, and patient outcomes. This compartmental approach facilitates a nuanced understanding of infection spread and control within healthcare environments.

**Model assumptions**

- Admissions are either uncolonised, colonised by ARB/ASB, or infected by ARB/ASB (community-acquired infections were incorporated).
- Infectiousness is uniform across colonised and infected patients, regardless of severity (mild or severe infections).
- Patients should become colonised before developing (progressing to) infection.
- Transmission occurs between ARB and Uncolonised, and ASB and uncolonised populations. ASB is different to uncolonised.
- Once CRE/MRSA colonised or infected, patients can revert to CRE/MRSA-negative in-hospital through regular antibiotic treatment following local guidelines, natural clearance, or decolonisation (intervention).
- Recovery is assumed post-discharge, if alive.
- We did not include different hospital specialities, apart from ICU/general wards within hospital dynamics.
- Length of stay is unaffected by colonisation, and it is assumed to be constant across uncolonised and colonised by CRE/CSE/MRSA/MSSA; only infections extend it and follows the distribution from the cohort data (sex-stratified).
- Mortality rates increase only with infection following our study cohort data, and it is assumed to be null among uncolonised and colonised by CRE/CSE/MRSA/MSSA individuals.
- We assumed a constant bed occupancy equivalent to between 80-90% total hospital beds (n=1,000 beds in the hospital).
- We assumed a fix proportion of women/men among the Uconlonised and colonised comparments representing sex-distribution in Chile. Distribution across sexes among infected populations followed our study cohort’s findings.
- We included a constant background rate that incorporates bacterial mutations.

All baseline conditions, parameters between compartments, and differential equations are detailed next.

**Table A1.** Baseline conditions within the compartments

| **Symbol** | **Description [Units]** | **Baseline value** | **Source** |
| --- | --- | --- | --- |
| **Population parameters:** *Staphylococcus aureus* | |  |  |
| N (t) | Population size [nº individuals] | N_(t=0)_=10,000 | Assumed as total hospitalised patients per day |
| U (t) | Uncolonised individuals [nº individuals] | U_(t=0)_= 0.7*N | Literature^2^ |
| C_R_ (t) | Patients colonised with MRSA [nº individuals] | C_(t=0)_= 0.12*N | Literature^3^ |
| C_S_ (t) | Patients colonised with MSSA [nº individuals] | C_(t=0)_= ((1-0.7)-0.12)*N | Assumed |
| I^R^_Mil_ (t) | Patients infected by MRSA experiencing mild symptoms [nº individuals] | I^R^_Mil(t=0)_= C_R_*(0.259)*(1-ICU admission MRSA) | Literature for progression^4^ and our study results for ICU admission |
| I^R^_Sev_ (t) | Patients infected by MRSA experiencing severe symptoms [nº individuals] | I^R^_Sev(t=0)_ =  C_R_*(0.259)*(ICU admission MRSA) | Literature for progression^4^ and our study results for ICU admission |
| I^S^_Mil_ (t) | Patients infected by MSSA experiencing mild symptoms [nº individuals] | I^S^_Mil (t=0)_= C_S_*(0.099)*(1-ICU admission MSSA) | Literature for progression^4^ and our study results for ICU admission |
| I^S^_Sev_ (t) | Patients infected by MSSA experiencing severe symptoms [nº individuals] | I^S^_Sev(t=0)_ =  C_S_*(0.099)*(ICU admission MSSA) | Literature for progression^4^ and our study results for ICU admission |
| D^R^ (t) | Deceased individuals [nº individuals] | 0 | Assumed |
| R^R^ (t) | Deceased individuals [nº individuals] | 0 | Assumed |
| D^S^ (t) | Deceased individuals [nº individuals] | 0 | Assumed |
| R^S^ (t) | Deceased individuals [nº individuals] | 0 | Assumed |
| **Population parameters:** Enterobacterales | |  |  |
| N (t) | Population size [nº individuals] | N_(t=0)_=10,000 | Assumed as total hospitalised patients per day |
| U (t) | Uncolonised individuals [nº individuals] | U_(t=0)_= 44.00%*N | Literature^5^. |
| C_R_ (t) | Patients colonised with CRE [nº individuals] | C_R(t=0)_= 14.45%*N | Literature^5^. |
| C_S_ (t) | Patients colonised with CSE [nº individuals] | C_S(t=0)_= 41.55%*N | Literature^5^. |
| I^R^_Mil_ (t) | Patients infected by CRE experiencing mild symptoms [nº individuals] | I^R^_Mil(t=0)_= C_R_*(0.09)*(*(1-ICU admission) | Literature for progression^6^ and our study results for ICU admissions. |
| I^R^_Sev_ (t) | Patients infected by CRE experiencing severe symptoms [nº individuals] | I^R^_Sev(t=0)_ =  C_R_*(0.09)*(ICU admission) | Literature for progression^6^ and our study results for ICU admissions. |
| I^S^_Mil_ (t) | Patients infected by CSE experiencing mild symptoms [nº individuals] | I^S^_Mil (t=0)_= C_S_*(0.04)*(1-ICU admission) | Literature for progression^7^ and our study results for ICU admissions. |
| I^S^_Sev_ (t) | Patients infected by CSE experiencing severe symptoms [nº individuals] | I^S^_Sev(t=0)_ =  C_S_*(0.04)*(ICU admission) | Literature^7^ and case study for ICU admissions. |
| D^R^ (t) | Deceased individuals, CRE [nº individuals] | 0 | Assumed |
| R^R^ (t) | Deceased individuals, CRE [nº individuals] | 0 | Assumed |
| D^S^ (t) | Deceased individuals, CSE [nº individuals] | 0 | Assumed |
| R^S^ (t) | Deceased individuals, CSE [nº individuals] | 0 | Assumed |

Notes: MINSAL= Ministry of Health Chile. CSE= Carbapenem-susceptible Enterobacterales. CRE= Carbapenem-resistant Enterobacterales. MSSA= Methicillin-susceptible Staphylococcus aureus. MRSA= Methicillin-resistant Staphylococcus aureus. U (Uncolonized). CR (Colonised by Resistant Bacteria). CS (Colonised by Susceptible Bacteria). IMR (Infected with Mild Resistant). ISR (Infected with Severe Resistant). IMS (Infected with Mild Susceptible). ISS (Infected with Severe Susceptible). RR (Recovered from Resistant Infection). RS (Recovered from Susceptible Infection). DR (Dead from Resistant Infection). DS (Dead from Susceptible Infection).

**Table A2.** Model parameters and variables with definitions and values

| **Symbol** | **Definition [Units]** | **Values** | **Source or Reference** |
| --- | --- | --- | --- |
| ***Staphylococcus aureus*** | |  |  |
| δ1, δ2 | Natural clearance from colonised by CR and CS states, respectively [1/unit time] [%]. | δ1=0.0016  δ2=0.0016 | Literature^8^ |
| $INF$ | Influx of new patient populations admitted to the hospital in each health state [rates * hospital admission/community acquired infections]. Measured as [1/unit time] [%]. | ${INF}_{women}^{U}$  ${INF}_{men}^{U}$  ${INF}_{women}^{CR}$  ${INF}_{men}^{CR}$  ${INF}_{women}^{CS}$  ${INF}_{men}^{CS}$  ${INF}_{women}^{IMR}$  ${INF}_{men}^{ISR}$  ${INF}_{women}^{IMS}$  ${INF}_{men}^{ISS}$ | These parameters are related to the influx of new admissions set to offset discharge at all ‘t’ times.  We assumed that a % of new hospital admissions are infected (*caIha*=0.001^9^) and distributed them equally throughout IMR, ISR IMS and ISS according to sex distributions (μ). |
| *Disch* | Discharge rates from asymptomatic uncolonised and colonised compartments (1/length of hospital stay) | ${Disch}_{U,men}$=1/6 ${Disch}_{U,women}$=1/6 ${Disch}_{CS,men}$=1/6 ${Disch}_{CS,women}$=1/6 ${Disch}_{CR,men}$=1/6 ${Disch}_{CR,women}$=1/6 | Calculated according to the average length of hospital stay in Chile. Literature ^10^. |
| μ | Proportion of women among specific populations (i.e., U, CR, CS, IMR, ISR, IMS, and ISS) [1/unit time] [%]. | μ0= 52%  μ1= 52%  μ2= 52%  μ3= 31.65%  μ4= 38.89%  μ5= 39.38%  μ6=44.87% | Literature for hospitalised women^5^ (we assume distribution of women is similar among U, C_R_ and C_S_) and our case-study’s data based on population cohort’s distributions among the infected populations. |
| τ1 | Transmission parameter between colonised, infected or uncolonized strains [1/unit time] [%]. | $\tau1$= 0.223 | Estimated. |
| $\psi$ | Percentage of patients exposed to vancomycin or penicillin [1/unit time] [%]. | $\psi_{men}$= 14.74%  $\psi_{women}$= 18.40% | Our case study based on vancomycin/penicillin consumption prior to blood culture. |
| c | Fitness cost. c reduces the transmission rate among resistant strains [1/unit time] [%]. | c= 9% [95% CI: 2-15%] | Literature^11^ |
| β1, β2 | Progression to the development of infection from colonisation among CR and CS states. It comprises the inverse of the mean time to infection in days multiplied by patients having clinical infection [(1/LOS)* percentage becoming infected or symptomatic]. | β1_men_= (1/21) *26%  β1_women_= (1/29) *26%  β2_men_= (1/11) *9.9%  β2_women_= (1/14)*9.9% | Literature^12-15^ and our case-study’s data based on specific length of hospital stays before infection (Table A12). |
| γ1, γ2, γ3, γ4 | Natural clearance of mild and severe infections among CR and CS states, respectively [1/unit time] [%]. | γ1= 0.001  γ2= 0.001  γ3= 0.001  γ4= 0.001 | Assumed. |
| ω1, ω2, ω3 ω4 | Mean time of infection considering length of hospital stays [1/length of hospital stay]. ω1_d_ and ω1_r_ are among IMR patients who either died or recovered, ω2_d_ and ω2_r_ are for ISR who either died or recovered, ω3_d_ and ω3_r_ are for IMS who either died or recovered, and ω4_d_ and ω4_r_ are for ISS who either died or recovered. | *ω1_d,men_=* (1/20) *ω1_r,men_=* (1/23) *ω1_d,women_=* (1/13) *ω1_r,women_=* (1/26) *ω2_d,men_=* (1/11) *ω2_r,men_=* (1/18) *ω2_d,women_=* (1/14) *ω2_r,women_=* (1/19) *ω3_d,men_=*(1/12) *ω3_r,men_=*(1/11) *ω3_d,women_=*(1/20) *ω3_r,women_=*(1/16) *ω4_d,men_=* (1/11) *ω4_r,men_=* (1/19) *ω4_d,women_=*(1/14) *ω4_r,women_=*(1/17) | Our case-study’s data based on estimated length of hospital stays (Table A13). |
| α1, α2 | Percentage of inpatients with CR or CS, respectively, progressing to severe infection in intensive care units [1/unit time] [%]. | α1_men_= 35.17% α1_women_= 41.96% α2_men_= 28.26% α2_women_= 34.38% | Our case-study’s data based on admissions to ICU (Figure A6). |
| ν1, ν2, ν3, ν4 | Recovery rates from infection, including IMR, ISR, IMS and ISS due to treatment received [1/unit time] [%]. | ν1_men_= (1- ζ1_men_) ν1_women_= (1- ζ1_women_) ν2_men_= (1- ζ2_men_) ν2_women_= (1- ζ2_women_) ν3_men_= (1- ζ3_men_) ν3_women_= (1- ζ3_women_) ν4_men_= (1- ζ4_men_) ν4_women_= (1- ζ4_women_) | Our case-study’s data based on recovery rates |
| ε1, ε2 | Progression from mild to severe infection from IMR and IMS, respectively [1/unit time] [%]. | ε1= 0.01 ε2= 0.01 | Assumed. |
| ζ1, ζ2, ζ3, ζ4 | Mortality rates from infection. ζ1 and ζ2 are mortality rates from mild and severe resistant infections, respectively. ζ3 and ζ4 are from mild and severe susceptible infections, respectively [1/unit time] [%]. | ζ1_men_= ζ3_men_*1.01 ζ1_women_= ζ3_men_*1.22 ζ2_men_= ζ3_men_*2.32 ζ2_women_= ζ3_men_*2.25 ζ3_men_=23.1% [Ref.] ζ3_women_= ζ3_men_*2.07 ζ4_men_= ζ3_men_*1.10 ζ4_women_= ζ3_men_*2.34 | Our case-study’s data based on mortality proportions and estimated hazard ratios across specific populations (Table A11). |
| b | Constant background rate that captures transmission from non-human sources, horizontal transmission, or de novo emergence [1/unit time] [number]. | b= 0.01 | Assumed. |
|  |  |  |  |
| **Enterobacterales** | | | |
| δ1, δ2 | Natural clearance from colonised by CR and CS states, respectively [1/unit time] [%]. | δ1=0.001  δ2= 0.001 | Assumed. |
| $INF$ | Influx of new patient populations admitted to the hospital in each health state [rates * hospital admission/community acquired infections]. Measured as [1/unit time] [%]. | ${INF}_{women}^{U}$  ${INF}_{men}^{U}$  ${INF}_{women}^{CR}$  ${INF}_{men}^{CR}$  ${INF}_{women}^{CS}$  ${INF}_{men}^{CS}$  ${INF}_{women}^{IMR}$  ${INF}_{men}^{ISR}$  ${INF}_{women}^{IMS}$  ${INF}_{men}^{ISS}$ | These parameters are related to the influx of new admissions set to offset discharge at all ‘t’ times.  We assumed that a % of new hospital admissions are infected (*caIha*=0.007^16^) and distributed them equally throughout IMR, ISR IMS and ISS according to sex distributions (μ). |
| *Disch* | Discharge rates from asymptomatic uncolonised and colonised compartments (1/length of hospital stay) | ${Disch}_{U,men}$=1/6 ${Disch}_{U,women}$=1/6 ${Disch}_{CS,men}$=1/6 ${Disch}_{CS,women}$=1/6 ${Disch}_{CR,men}$=1/6 ${Disch}_{CR,women}$=1/6 | Calculated according to the average length of hospital stay in Chile. Literature ^10^. |
| μ | Proportion of women among specific populations (i.e., U, CR, CS, IMR, ISR, IMS, and ISS) [1/unit time] [%]. | μ0=52%  μ1= 52%  μ2= 52%  μ3= 33.94%  μ4= 32.80%  μ5= 50.0%  μ6= 44.4% | Literature for hospitalised women^5^ (we assume distribution of women is similar among U, C_R_ and C_S_) and our case-study’s data based on population cohort’s distributions among the infected populations. |
| τ1 | Transmission parameter between colonised, infected or uncolonized strains [1/unit time] [%]. | $\tau1$= 0.399 | Estimated. |
| ψ | Percentage of patients exposed to carbapenems (ertapenem, meropenem and imipenem) before blood culture [1/unit time] [%]. | $\psi_{men}$=22.25%  $\psi_{women}$=20.26% | Our case study based on carbapenem consumption prior to blood culture. |
| c | Fitness cost. c reduces the transmission rate among resistant strains [1/unit time] [%]. | c= (1-0.927) | Literature^17^. |
| β1, β2 | Progression to the development of infection from colonisation among CR and CS states. It comprises the inverse of the mean time to infection in days multiplied by patients having clinical infection [(1/LOS)* percentage becoming infected or symptomatic]. | β1_men_= (1/22) *21.3%  β1_woen_= (1/27)*21.3%  β2_men_= (1/20)*3.4%  β2_women_= (1/17)*3.4% | Literature^18-20^ our case-study’s data based on specific length of hospital stays before infection (Table A12). |
| γ1, γ2, γ3, γ4 | Natural clearance of mild and severe infections among CR and CS states, respectively [1/unit time] [%]. | γ1= 0.001  γ2= 0.001  γ3= 0.001  γ4= 0.001 | Assumed. |
| ω1, ω2, ω3 ω4 | Mean time of infection considering length of hospital stays [1/length of hospital stay]. ω1_d_ and ω1_r_ are among IMR patients who either died or recovered, ω2_d_ and ω2_r_ are for ISR who either died or recovered, ω3_d_ and ω3_r_ are for IMS who either died or recovered, and ω4_d_ and ω4_r_ are for ISS who either died or recovered. | *ω1_d,men_=* (1/21) *ω1_r,men_=* (1/26) *ω1_d,women_=* (1/31) *ω1_r,women_=* (1/30) *ω2_d,men_=* (1/7) *ω2_r,men_=* (1/20) *ω2_d,women_=* (1/20) *ω2_r,women_=* (1/23) *ω3_d,men_=*(1/12) *ω3_r,men_=*(1/20) *ω3_d,women_=*(1/10) *ω3_r,women_=*(1/18) *ω4_d,men_=* (1/11) *ω4_r,men_=* (1/14) *ω4_d,women_=*(1/9) *ω4_r,women_=*(1/15) | Our case-study’s data based on specific length of hospital stays (Table A13). |
| α1, α2 | Percentage of inpatients with CR or CS, respectively, progressing to severe infection in intensive care units [1/unit time] [%]. | α1_men_= 45.85% α1_women_= 42.83% α2_men_= 38.32% α2_women_= 35.48% | Our case-study’s data based on admissions to ICU (Figure A5). |
| ν1, ν2, ν3, ν4 | Recovery rates from infection, including IMR, ISR, IMS and ISS due to treatment received [1/unit time] [%]. | ν1_men_= (1- ζ1_men_) ν1_women_= (1- ζ1_women_) ν2_men_= (1- ζ2_men_) ν2_women_= (1- ζ2_women_) ν3_men_= (1- ζ3_men_) ν3_women_= (1- ζ3_women_) ν4_men_= (1- ζ4_men_) ν4_women_= (1- ζ4_women_) | Our case-study’s data based on recovery rates |
| ε1, ε2 | Progression from mild to severe infection from IMR and IMS, respectively [1/unit time] [%]. | ε1= 0.01 ε2= 0.01 | Assumed. |
| ζ1, ζ2, ζ3, ζ4 | Mortality rates from infection. ζ1 and ζ2 are mortality rates from mild and severe resistant infections, respectively. ζ3 and ζ4 are from mild and severe susceptible infections, respectively [1/unit time] [%]. | ζ1_men_= ζ3_men_*1.80 ζ1_women_= ζ3_men_*0.55 ζ2_men_= ζ3_men_*1.30 ζ2_women_= ζ3_men_*2.40 ζ3_men_= 22.8% [Ref.] ζ3_women_= ζ3_men_*0.81 ζ4_men_= ζ3_men_*1.62 ζ4_women_= ζ3_men_*2.23 | Our case-study’s data based on mortality proportions and estimated hazard ratios across specific populations (Table A11). |
| b | Constant background rate that captures transmission from non-human sources, horizontal transmission, or de novo emergence [1/unit time] [number]. | b=0.01 | Assumed. |

*Notes:* Ref.= References. CRE= Carbapenem-resistant Enterobacterales. CSE= Carbapenem-susceptible Enterobacterales. MRSA= Methicillin-resistant *Staphylococcus aureus*. MSSA= Methicillin-susceptible *Staphylococcus aureus*. U (Uncolonized). CR (Colonised by Resistant Bacteria). CS (Colonised by Susceptible Bacteria). IMR (Infected with Mild Resistant). ISR (Infected with Severe Resistant). IMS (Infected with Mild Susceptible). ISS (Infected with Severe Susceptible). RR (Recovered from Resistant Infection). RS (Recovered from Susceptible Infection). DR (Dead from Resistant Infection). DS (Dead from Susceptible Infection). Ref.= reference term.

**Text A1. Definition of parameters and differential Equations.**

Differential equations

Where subindex p=pathogen (i.e., *Staphylococcus aureus* or Enterobacterales), t= time in days, and U (Uncolonized). CR (Colonised by Resistant Bacteria). CS (Colonised by Susceptible Bacteria). IMR (Infected with Mild Resistant). ISR (Infected with Severe Resistant). IMS (Infected with Mild Susceptible). ISS (Infected with Severe Susceptible). RR (Recovered from Resistant Infection). RS (Recovered from Susceptible Infection). DR (Dead from Resistant Infection). DS (Dead from Susceptible Infection).

$$\frac{\boldsymbol{d}\boldsymbol{U}_{\boldsymbol{p, men}}}{\boldsymbol{dt}}={\delta1}_{p}*{CR}_{p,men}+{\delta2}_{p}*{CS}_{p,men}+{INF}_{p,men}^{U}-{\left( 1-\mu0 \right)\mathrm{FOCefflux}}_{U, p}-{U_{p,men}*Disch}_{U,p,men}{+\psi}_{p,men}*{CS}_{p,men}{+\psi tr}_{p,men}*{CR}_{p,men}$$

$$\frac{\boldsymbol{d}\boldsymbol{U}_{\boldsymbol{p, women}}}{\boldsymbol{dt}}={\delta1}_{p}*{CR}_{p,women}+{\delta2}_{p}*{CS}_{p,women}+{INF}_{p,women}^{U}-{(\mu0)(FOCefflux}_{U, p})-{U_{p,women}*Disch}_{U,p,women}{+\psi}_{p,women}*{CS}_{p,women}{+\psi tr}_{p,women}*{CR}_{p,women}$$

$$\frac{\boldsymbol{d}\boldsymbol{CR}_{\boldsymbol{p,men}}}{\boldsymbol{dt}}={-\delta1}_{p}*{CR}_{p,men}-{\beta1}_{p}*{CR}_{p,men}+{\gamma1}_{p}*{IMR}_{p,men}+{\gamma2}_{p}*{ISR}_{p,men}+{INF}_{p,men}^{CR}+{\left( 1-\mu1 \right)(FOC}_{CR, p})-{{CR}_{p,men}*Disch}_{CR,p,men}-{\psi tr}_{p,men}*{CR}_{p,men}$$

$$\frac{\boldsymbol{d}\boldsymbol{CR}_{\boldsymbol{p,women}}}{\boldsymbol{dt}}={-\delta1}_{p}*{CR}_{p,women}-{\beta1}_{p}*{CR}_{p,women}+{\gamma1}_{p}*{IMR}_{p,women}+{\gamma2}_{p}*{ISR}_{p,women}+{INF}_{p,women}^{CR}+{(\mu1)(FOC}_{CR, p})-{{CR}_{p,women}*Disch}_{CR,p,women}-{\psi tr}_{p,women}*{CR}_{p,women}$$

$$\frac{\boldsymbol{d}\boldsymbol{CS}_{\boldsymbol{p,men}}}{\boldsymbol{dt}}={-\delta2}_{p}*\mathrm{CS}_{p,men} -{\beta2}_{p}*{CS}_{p,men} + {\gamma3}_{p}*{IMS}_{p,men}+{\gamma4}_{p}*{ISS}_{p,men}+{INF}_{p,men}^{CS}+{(1-\mu2)(FOC}_{CS, p})-{{CS}_{p,men}*Disch}_{CS,p,men}-\psi_{p,men}*{CS}_{p,men}$$

$$\frac{\boldsymbol{d}\boldsymbol{CS}_{\boldsymbol{p,women}}}{\boldsymbol{dt}}={-\delta2}_{p}*\mathrm{CS}_{p,women}-{\beta2}_{p}*{CS}_{p,women}+ {\gamma3}_{p}*{IMS}_{p,women}+{\gamma4}_{p}*{ISS}_{p,women}+{INF}_{p,women}^{CS}+{\left( \mu2 \right)(FOC}_{CS, p})-{{CS}_{p,women}*Disch}_{CS,p,women}-\psi_{p,women}*{CS}_{p,women}$$

$$\frac{\boldsymbol{d}\boldsymbol{IMR}_{\boldsymbol{p,men}}}{\boldsymbol{dt}}={\beta1}_{p}*{CR}_{p,men}*\left( 1-\alpha1_{p,men} \right)-{\gamma1}_{p}*{IMR}_{p,men}- {\left( {\omega1r}_{p,men} \right)\nu1}_{p,men}*{IMR}_{p,men}- {\varepsilon1}_{p}*{IMR}_{p,men}-{\left( {\omega1d}_{p,men} \right)\zeta1}_{p,men}*{IMR}_{p,men}+{INF}_{p,men}^{IMR}$$

$$\frac{\boldsymbol{d}\boldsymbol{IMR}_{\boldsymbol{p,women}}}{\boldsymbol{dt}}={\beta1}_{p}*{CR}_{p,women}*\left( 1-\alpha1_{p,women} \right)-{\gamma1}_{p}*{IMR}_{p,women}- {\left( {\omega1r}_{p,women} \right)\nu1}_{p,women}*{IMR}_{p,women}- {\varepsilon1}_{p}*{IMR}_{p,women}-{\left( {\omega1d}_{p,women} \right)\zeta1}_{p,women}*{IMR}_{p,women}+{INF}_{p,women}^{IMR}$$

$$\frac{\boldsymbol{d}\boldsymbol{ISR}_{\boldsymbol{p,men}}}{\boldsymbol{dt}}={\beta1}_{p}*{CR}_{p,men}*\left( \alpha1_{p,men} \right)-{\gamma2}_{p}*{ISR}_{p,men}-{\left( {\omega2r}_{p,men} \right)\nu2}_{p,men}*{ISR}_{p,men}+{\varepsilon1}_{p}*{IMR}_{p,men}-\left( {\omega2d}_{p,men} \right){\zeta2}_{p,men}*{ISR}_{p,men}+{INF}_{p,men}^{ISR}$$

$$\frac{\boldsymbol{d}\boldsymbol{ISR}_{\boldsymbol{p,women}}}{\boldsymbol{dt}}={\beta1}_{p}*{CR}_{p,women}*\left( \alpha1_{p,women} \right)-{\gamma2}_{p}*{ISR}_{p,women}-{\left( {\omega2r}_{p,women} \right)\nu2}_{p,women}*{ISR}_{p,women}+{\varepsilon1}_{p}*{IMR}_{p,women}-\left( {\omega2d}_{p,women} \right){\zeta2}_{p,women}*{ISR}_{p,women}+{INF}_{p,women}^{ISR}$$

$$\frac{\boldsymbol{d}\boldsymbol{IMS}_{\boldsymbol{p,men}}}{\boldsymbol{dt}}={\beta2}_{p}*{CS}_{p,men}*\left( 1-\alpha2_{p,men} \right)-{\gamma3}_{p,s}*{IMS}_{p,s}- \left( {\omega3r}_{p,men} \right){\nu3}_{p,men}*{IMS}_{p,men}- \varepsilon2*{ISS}_{p,men}-\left( {\omega3d}_{p,men} \right){\zeta3}_{p,men}*{IMS}_{p,men}+{INF}_{p,men}^{IMS}$$

$$\frac{\boldsymbol{d}\boldsymbol{IMS}_{\boldsymbol{p,women}}}{\boldsymbol{dt}}={\beta2}_{p}*{CS}_{p,women}*\left( 1-\alpha2_{p,women} \right)-{\gamma3}_{p,s}*{IMS}_{p,s}- \left( {\omega3r}_{p,women} \right){\nu3}_{p,women}*{IMS}_{p,women}- \varepsilon2*{ISS}_{p,women}-\left( {\omega3d}_{p,women} \right){\zeta3}_{p,women}*{IMS}_{p,women}+{INF}_{p,women}^{IMS}$$

$$\frac{\boldsymbol{d}\boldsymbol{ISS}_{\boldsymbol{p,men}}}{\boldsymbol{dt}}={\beta2}_{p}*{CS}_{p,men}*\left( \alpha2_{p,men} \right)- \gamma4*{ISS}_{p,men}- \left( {\omega4r}_{p,men} \right){\nu4}_{p,men}*{ISS}_{p,men}+ \varepsilon2*{ISS}_{p,men}- \left( {\omega4d}_{p,men} \right){\zeta4}_{p,men}* {ISS}_{p,men}+{INF}_{p,men}^{ISS}$$

$$\frac{\boldsymbol{d}\boldsymbol{ISS}_{\boldsymbol{p,women}}}{\boldsymbol{dt}}={\beta2}_{p}*{CS}_{p,women}*\left( \alpha2_{p,women} \right)- \gamma4*{ISS}_{p,women}- \left( {\omega4r}_{p,women} \right){\nu3}_{p,women}*{ISS}_{p,women}+\varepsilon2*{ISS}_{p,women}- \left( {\omega4d}_{p,s} \right){\zeta4}_{p,women}* {ISS}_{p,women}+{INF}_{p,women}^{ISS}$$

$$\frac{\boldsymbol{d}\boldsymbol{RR}_{\boldsymbol{p,men}}}{\boldsymbol{dt}}=({\omega1r}_{p,men}){\nu1}_{p,men}*{IMR}_{p,men}+ ({\omega2r}_{p,men}){\nu2}_{p,men}*{ISR}_{p,men}$$

$$\frac{\boldsymbol{d}\boldsymbol{RR}_{\boldsymbol{p,women}}}{\boldsymbol{dt}}=({\omega1r}_{p,women}){\nu1}_{p,women}*{IMR}_{p,women}+ ({\omega2r}_{p,women}){\nu2}_{p,women}*{ISR}_{p,women}$$

$$\frac{\boldsymbol{d}\boldsymbol{RS}_{\boldsymbol{p,men}}}{\boldsymbol{dt}}= ({\omega3r}_{p,men}){\nu3}_{p,men}*{IMS}_{p,men}+ ({\omega4r}_{p,men}){\nu4}_{p,men}*{ISS}_{p,men}$$

$$\frac{\boldsymbol{d}\boldsymbol{RS}_{\boldsymbol{p,women}}}{\boldsymbol{dt}}= ({\omega3r}_{p,women}){\nu3}_{p,women}*{IMS}_{p,women}+ ({\omega4r}_{p,women}){\nu4}_{p,women}*{ISS}_{p,women}$$

$$\frac{\boldsymbol{d}\boldsymbol{DR}_{\boldsymbol{p,men}}}{\boldsymbol{dt}}= ({\omega1d}_{p,men}){\zeta1}_{p,men}*{IMR}_{p,men}+ ({\omega2d}_{p,men}){\zeta2}_{p,men}*{ISR}_{p,men}$$

$$\frac{\boldsymbol{d}\boldsymbol{DR}_{\boldsymbol{p,women}}}{\boldsymbol{dt}}= ({\omega1d}_{p,women}){\zeta1}_{p,women}*{IMR}_{p,women}+ ({\omega2d}_{p,women}){\zeta2}_{p,women}*{ISR}_{p,women}$$

$$\frac{\boldsymbol{d}\boldsymbol{DS}_{\boldsymbol{p,men}}}{\boldsymbol{dt}}= ({\omega3d}_{p,men}){\zeta3}_{p,men}*{IMS}_{p,men}+ ({\omega4d}_{p,men}){\zeta4}_{p,men}*{ISS}_{p,men}$$

$$\frac{\boldsymbol{d}\boldsymbol{DS}_{\boldsymbol{p,women}}}{\boldsymbol{dt}}= ({\omega3d}_{p,women}){\zeta3}_{p,women}*{IMS}_{p,women}+ ({\omega4d}_{p,women}){\zeta4}_{p,women}*{ISS}_{p,women}$$

Equations for the force of colonisation among different health states:

$\mathrm{FOC}_{CR, p}$**= (**$\tau1*(1-c)*\sum_{s=men}^{women} {((CR}_{p,s}+{IMR}_{p,s}+{ISR}_{p,s})*U_{p,s}))+ b)/N$

$\mathrm{FOC}_{CS, p}$**=** $\boldsymbol{(}\tau1*\sum_{s=men}^{women} {(CS}_{p,s}+{IMS}_{p,s}+{ISS}_{p,s}*U_{p,s}))/N$

$\mathrm{FOCefflux}_{U, p}=\mathrm{FOC}_{CS, p}$**+**$\mathrm{FOC}_{CR, p}$

# III. Intervention details and economic costs

Interventions followed a testing scheme (chromogenic agar, chromogenic agar enriched with oxacillin or carbapenems, and PCR) plus decolonisation treatment or isolation, or pre-emptive isolation with no test. Decolonisation for MRSA involved: mupirocin (20mg, 3 times per day for 5 days; equivalent to $0.60 per day) was administered by a nurse (average wage/hour= $11.9) but considering half an hour of their time daily ($5.9). Total costs per patient/treatment is $32.5 (or $6.5 per patient/day). For CRE eradication, we employed a selective digestive decontamination (SDD) treatment in carriers^21,22^, comprising gentamicin (80 mg) and colistin (100 mg) 4 times daily for 7 days, orally or through a nasogastric tube. This is equivalent to $72.88 per day.

A patient in isolation would receive 12 nurse visits daily, every two hours, and two additional physician visits. Assuming each visit lasts 10 minutes and considering the doctor's hourly wage of $34.5, the total daily cost for the extra time spent by nurses and physicians is estimated at $35.3 per patient/day. For each visit, both nurses and physicians utilise a pair of gloves and a gown ($0.5 per visit). The total isolation cost per patient/day is $42.3.

See Tables below for a description of parameters and cost utilised in our model.

**Table A3.** Strategy schemes

| **Strategy** | **Screening method, time results** | **Patients screened** | **Patients targeted** | **Procedure and timing** |
| --- | --- | --- | --- | --- |
| Testing + decolonisation | **(i)** Chromogenic agar test, 48 hours + processing | All new admissions and sex-specific | CRE+ and MRSA+ | Testing using any of the screening methods considering their sensitivity/specificity^23-33^ and turnaround time^23-27,31,34-36^ (see Supplementary Tables 3-4) plus decolonisation treatment as detailed below.  MRSA decolonisation: Mupirocin (20mg, 3 times per day for 5 days^37,38^) was administered by a nurse.  CRE decolonisation: Selective digestive decontamination (SDD) treatment in carriers^21,22,38-40^, comprising gentamicin (80 mg) and colistin (100 mg) 4 times daily for 7 days, orally or through a nasogastric tube. |
|  | **(ii)** Chromogenic gar test enriched with salt and oxacillin OR carbapenems, 24 hours + processing |  |  |  |
|  | **(iii)** PCR, 24 hours |  |  |  |
| Testing + isolation (contact precaution) | **(i)** Chromogenic agar test, 48 hours + processing | All new admissions | CRE+ and MRSA+ | Testing as detailed above plus contact precaution measures comprising constant patient monitoring and the use of gloves and gown by medical staff. A patient in isolation would receive 12 nurse visits daily, every two hours, and two additional physician visits. |
|  | **(ii)** Chromogenic gar test enriched with salt and oxacillin OR carbapenems, 24 hours + processing |  |  |  |
|  | **(iii)** PCR, 24 hours |  |  |  |
| Pre-emptive isolation (contact precaution) | No test | All new admissions and sex-specific | All | Equivalent to contact precaution measures but pre-emptively at admission with no test. |

Notes: PCR= polymerase chain reaction. CRE= Carbapenem-resistant Enterobacterales. MRSA= Methicillin-resistant *Staphylococcus aureus*.

**Table A4.** Additional parameters for tests and interventions including epidemiological effects

| **Parameters and subitems** | **Value** | **Source** |
| --- | --- | --- |
| **I. Chromogenic agar (48 hrs + processing)** |  |  |
| Sensitivity | 82.6% | ^28-31,33^ |
| Specificity | 83.1% | ^28-31,33^ |
| Turnaround time | 3 days | ^31,34^ |
| **IIa. Chromogenic agar early results enriched with salt and oxacillin (24 hrs + processing)** |  |  |
| Sensitivity | 62.2% | ^28-33^ |
| Specificity | 97.1% | ^28-33^ |
| Turnaround time | 2 days | ^31,34^ |
| **IIb. Chromogenic agar early results enriched with carbapenems (24 hrs + processing)** |  |  |
| Sensitivity | 90% | ^23-27^ |
| Specificity | 93% | ^23-27^ |
| Turnaround time | 2 days | ^23-27^ |
| **IIIa. Polymerase chain reaction (PCR) for MRSA** |  |  |
| Sensitivity | 88.4% | ^31,33,41-45^ |
| Specificity | 83.8% | ^31,33,41-45^ |
| Turnaround time | 1 day | ^31,34^ |
| **IIIb. Polymerase chain reaction (PCR) for CRE** |  |  |
| Sensitivity | 100% | ^35,36^ |
| Specificity | 100% | ^35,36^ |
| Turnaround time | 1 day | ^35,36^ |
| **IV. Isolation parameters (contact precaution)** |  |  |
| Reduction in transmission from patients under contact precautions with MRSA | 36.5% | ^37^ |
| Reduction in transmission from patients under contact precautions with CRE | 35.0% | ^38^ |
| **V. Efficiency of decolonisation treatment in clearing resistance** |  |  |
| Efficiency of MRSA decolonisation | 53% | ^31,46^ |
| Efficiency of CRE SDD treatment | 26% | ^21,22,39,40^ |
| Effect on probability of progression /self-infection (percentage reduction) mupirocin for MRSA | 33% | ^47^ |
| Effect on probability of progression /self-infection (percentage reduction) chlorhexidine bathing for MRSA | 31% | ^31^ |
| Effect on probability of progression /self-infection (percentage reduction) SDD for CRE | 4.1% | ^39^ |

Notes: MRSA= Methicillin-resistant *Staphylococcus aureus*. CRE= Carbapenem-resistant Enterobacterales.

**Table A5.** Cost components of the interventions and bed-days in hospital settings

| **Cost component** | **Cost** | **Source** | **Additional information** |
| --- | --- | --- | --- |
| **I. Hospital bed-days** |  |  |  |
| General ward | $50 | FONASA^48^ | Cost per day |
| General ward with intermediate care | $92 | FONASA^48^ | Cost per day |
| Intensive care unit | $218 | FONASA^48^ | Cost per day |
| **II. Drug costs** |  |  |  |
| Gentamicin | $0.02 | CENABAST^49^ | Cost per 80mg/ampoule |
| Colistin | $18.2 | CENABAST^49^ | Cost per 100mg |
| Mupirocin 2% ointment | $0.20 | CENABAST^49^ | Cost per gram (20mg of mupirocin) |
| Chlorhexidine 2% soluble | $0.24 | CENABAST^49^ | Cost per 200ml |
| **III.** **Diagnostic tests** |  |  |  |
| Real-time PCR for viruses and bacteria (swab included) | $33 | FONASA^48^ | Cost per test |
| Chromogenic agar 24 hours mannitol salt agar with oxacillin | $13.6 | Mercado publico^50^ | Cost per test/plate |
| Chromogenic agar 48 hours (MCConkey) | $10.2 | Mercado publico^50^ | Cost per test/plate |
| Automated blood culture with antimicrobial susceptibility test included | $16.9 | FONASA^48^ | Cost per test |
| **IV.** **Intervention costs** |  |  |  |
| Contact precaution cost per day | $42.3 | Calculated | Cost per day/patient |
| Use of gloves and gown | $0.5 | Calculated^48^ | Cost per day/patient |
| Nurse wage | $11.9 | Calculated^51^ | Cost per hour |
| Doctor wage | $34.5 | Calculated^52^ | Cost per hour |
| **VI. Health utilities** |  |  |  |
| 18-65 years old utility | 0.92 | ^53^ | - |
| Disutility for hospitalization in the ICU | −0.34 | ^54^ | - |
| Infected inpatient, not ICU | 0.64 | ^55,56^ | - |
| Recovered patient from severe ICU infection | 0.74 | ^57^ | - |
| Dead | 0 | - | Assumed |

Notes: FONASA: Fondo Nacional de Salud, the financial entity entrusted to collect, manage and distribute state funds for health in Chile. CENABAST: Institution that manages the purchasing processes mandated by the Ministry of Health. DDD= Daily defined dosis. DDDs followed the World Health Organization standards.

# IV. Descriptive statistics among populations of interest

**Table A6.** Descriptive statistics among patients presenting with bloodstream infections produced by carbapenem-susceptible (CSE) or carbapenem-resistant (CRE) Enterobacterales.

| **Variables** | **Male** (n=278) | | | | | | | **Female** (n=191) | | | | |
| --- | --- | --- | --- | --- | --- | --- | --- | --- | --- | --- | --- | --- |
|  | **CSE** (n=122) | | **CRE** (n=156) | | | χ2 or T-tests p-value | | **CSE** (n=113) | | **CRE** (n=78) | | χ2 or T-tests |
|  | Mean (%) | 95%CI | Mean (%) | 95%CI | |  |  | Mean (%) | 95%CI | Mean (%) | 95%CI | p-value |
| **Outcome variables** |  |  |  |  | |  |  | |  |  |  |  |
| Overall mortality (%) | 23.77% | 17—32 | 30.13% | 23—38 | | 0.239 | 23.01% | | 16—32 | 30.77% | 21—42 | 0.233 |
| Full hospital LOS (days) | 38.01 | 5—95 | 42.65 | 9—108 | | 0.358 | 25.91 | | 1—82 | 49.85 | 6—143 | <0.001 |
| LOS before BC (days) | 14.19 | 0—46 | 18.63 | 0—61 | | 0.064 | 8.51 | | 0—30 | 21.97 | 0—69 | <0.001 |
| LOS after BC (days) | 22.87 | 2—71 | 24.28 | 2—73 | | 0.701 | 17.64 | | 1—57 | 29.96 | 1—108 | 0.001 |
| Full ICU admission (%) | 54.10% | 45—63 | 58.33% | 50—66 | | 0.482 | 47.79% | | 38—57 | 62.82% | 51—74 | 0.041 |
| ICU admission (%) before BC | 8.20% | 4—15 | 0.64% | 0—4 | | 0.001 | 7.96% | | 4—15 | 2.56% | 0—9 | 0.117 |
| ICU admission (%) after BC | 36.89% | 28—46 | 53.85% | 46—62 | | 0.005 | 31.86% | | 23—41 | 52.56% | 41—64 | 0.004 |
| Full ICU LOS (days) | 9.42 | 0—46 | 15.13 | 0—54 | | 0.012 | 6.54 | | 0—38 | 20.04 | 0—93 | <0.001 |
| ICU LOS after BC (days) | 8.87 | 0—46 | 14.85 | 0—54 | | 0.008 | 5.96 | | 0—38 | 19.64 | 0—93 | <0.001 |
| **Independent variables** |  |  |  |  | |  |  | |  |  |  |  |
| Age (years) | 62.65 | 37—86 | 60.69 | 32—87 | | 0.289 | 62.92 | | 32—84 | 56.45 | 26—84 | 0.010 |
| Hospitalisation in last three months (%) | 21.74% | 15—30 | 19.85% | 14—28 | | 0.715 | 24.32% | | 17—33 | 13.43% | 6—24 | 0.081 |
| Antibiotic consumption in last three months (%) | 8.74% | 4—16 | 15.27% | 10—23 | | 0.134 | 13.13% | | 7—21 | 4.92% | 1—14 | 0.094 |
| CCI (mean) | 3.37 | 0—900 | 3.11 | 0—9 | | 0.448 | 3.21 | | 0—7 | 2.94 | 0—9 | 0.450 |
| Null, CCI = 0 (%) | 13.93% | 8—21 | 14.10% | 9—21 | | 0.968 | 9.73% | | 5—17 | 23.08% | 14—34 | 0.011 |
| Mild, CCI = 1 or 2 (%) | 31.97% | 24—41 | 38.46% | 31—47 | | 0.263 | 27.43% | | 19—37 | 32.05% | 22—44 | 0.493 |
| Moderate, CCI = 3 or 4 (%) | 27.87% | 20—37 | 21.15% | 15—28 | | 0.195 | 38.05% | | 29—48 | 20.51% | 12—31 | 0.010 |
| Severe, CCI ≥ 5 (%) | 26.23% | 19—35 | 26.28% | 20—34 | | 0.992 | 24.78% | | 17—34 | 24.36% | 15—35 | 0.948 |
| Source of the BSI |  |  |  |  | |  |  | |  |  |  |  |
| Primary (%) | 26.73% | 18—36 | 38.69% | 30—47 | | 0.054 | 31.46% | | 22—42 | 43.28% | 31—56 | 0.131 |
| Catheter (%) | 6.93% | 3—14 | 13.87% | 9—21 | | 0.091 | 14.61% | | 8—24 | 14.93% | 7—26 | 0.956 |
| Pneumonia/respiratory (%) | 29.70% | 21—40 | 14.60% | 9—22 | | 0.005 | 10.11% | | 5—18 | 13.43% | 6—24 | 0.524 |
| Gastrointestinal (%) | 9.90% | 5—17 | 14.60% | 9—22 | | 0.283 | 22.47% | | 14—33 | 10.45% | 4—20 | 0.050 |
| Abdomen (%) | 22.77% | 15—32 | 13.87% | 9—21 | | 0.076 | 14.61% | | 8—24 | 16.42% | 8—27 | 0.758 |
| Bones and joints (%) | 1.98% | 0—7 | 1.46% | 0—5 | | 0.759 | 3.37% | | 1—10 | 0.00% | 0—5 | 0.131 |
| Skin and soft tissue (%) | 0.99% | 0—5 | 2.19% | 0—6 | | 0.479 | 3.37% | | 1—10 | 1.49% | 0—8 | 0.466 |
| Meningitis (%) | 0.99% | 0—5 | 0.73% | 0—4 | | 0.829 | 0.00% | | 0—4 | 0.00% | 0—5 | 0.999 |
| Community-acquired infection (%) | 32.77% | 24—42 | 13.64% | 9—20 | | <0.001 | 42.86% | | 34—53 | 15.58% | 8—26 | <0.001 |
| Indwelling catheter (%) | 37.70% | 29—47 | 53.85% | 46—62 | | 0.007 | 30.97% | | 23—40 | 50.00% | 38—62 | 0.008 |
| Kidney therapy before BC (%) | 2.65% | 1—8 | 6.57% | 3—12 | | 0.151 | 5.15% | | 2—12 | 0.00% | 0—5 | 0.062 |
| Transfer from another hospital (%) | 18.03% | 12—26 | 14.84% | 10—21 | | 0.476 | 18.02% | | 11—26 | 12.82% | 6—22 | 0.338 |
| ID specialist consultation (%) | 31.71% | 22—43 | 77.95% | 70—85 | | <0.001 | 16.42% | | 8—27 | 77.05% | 65—87 | <0.001 |
| Mechanical ventilation before BC (%) | 6.56% | 3—13 | 6.41% | 3—11 | | 0.961 | 5.31% | | 2—11 | 2.56% | 0—9 | 0.354 |
| Mechanical ventilation after BC (%) | 28.69% | 21—38 | 48.08% | 40—56 | | 0.001 | 17.70% | | 11—26 | 53.85% | 42—65 | <0.001 |
| Surgery previous BC (%) | 1.64% | 0—6 | 1.28% | 0—5 | | 0.805 | 0.88% | | 0—5 | 0.00% | 0—5 | 0.408 |
| Surgery after BC (%) | 6.56% | 3—13 | 14.10% | 9—21 | | 0.044 | 4.42% | | 1—10 | 6.41% | 2—14 | 0.547 |
| **Antibiotic consumption in daily defined doses ‘DDDs’ per treatment course after BC** | | | | |  |  |  |  |  |  |  |  |
| Total consumption (DDDs per 1,000 bed-days) | 179.71 | 0—568 | 340.20 | 0—1112 | | <0.001 | 204.65 | | 0—794 | 232.98 | 0—868 | 0.001 |
| Carbapenems (DDDs per 1,000 bed-days) | 17.93 | 0—87 | 53.54 | 0—165 | | <0.001 | 8.50 | | 0—57 | 17.69 | 0—100 | <0.001 |
| Cephalosporins (DDDs per 1,000 bed-days) | 64.28 | 0—175 | 85.26 | 0—288 | | 0.011 | 62.93 | | 0—340 | 60.41 | 0—167 | 0.266 |
| Macrolides (DDDs per 1,000 bed-days) | 3.50 | 0—0 | 7.41 | 0—82 | | 0.234 | 1.13 | | 0—0 | 9.01 | 0—67 | 0.408 |
| Fluoroquinolones (DDDs per 1,000 bed-days) | 9.18 | 0—87 | 8.65 | 0—82 | | 0.995 | 7.94 | | 0—113 | 2.67 | 0—0 | 0.743 |
| Aminoglycoside (DDDs per 1,000 bed-days) | 15.30 | 0—87 | 36.66 | 0—165 | | 0.001 | 3.97 | | 0—0 | 10.68 | 0—67 | 0.021 |
| Tetracyclines (DDDs per 1,000 bed-days) | 0.00 | 0—0 | 3.29 | 0—0 | | 0.039 | 0.00 | | 0—0 | 1.00 | 0—0 | 0.650 |
| Penicillin (DDDs per 1,000 bed-days) | 24.92 | 0—131 | 45.72 | 0—247 | | 0.017 | 72.56 | | 0—397 | 18.02 | 0—100 | 0.278 |
| Glycopeptides (DDDs per 1,000 bed-days) | 20.11 | 0—131 | 53.13 | 0—206 | | <0.001 | 34.01 | | 0—170 | 85.11 | 0—300 | <0.001 |
| LOT (days) | 17.17 | 0—57 | 35.13 | 0—99 | | <0.001 | 14.29 | | 0—61 | 30.09 | 0—80 | <0.001 |
| NOA (number) | 2.57 | 0—8 | 4.80 | 0—13 | | <0.001 | 2.31 | | 0—10 | 4.32 | 0—16 | <0.001 |

Notes: ARB= Antibiotic resistance. ASB= Antibiotic sensitive. BSI= Bloodstream infection. LOT= length of therapy defined as number of days a patient receives any antibiotic. NOA= Number of antibiotics used for treating a patient. CCI= Charlson comorbidity index. 95% CI for proportion variables were estimated49. BC= index Blood culture. ID= Infectious disease. ICU= Intensive care unit. LOS= Length of hospital stay. bχ2 or T-test were employed according to each variable’s distribution (α=0.05).

**Table A7.** Descriptive statistics among patients presenting with bloodstream infections produced by methicillin-susceptible (MSSA) or methicillin-resistant (MRSA) *Staphylococcus aureus*.

| **Variables** | **Male** (n=247) | | | | | | | **Female** (n=157) | | | | |
| --- | --- | --- | --- | --- | --- | --- | --- | --- | --- | --- | --- | --- |
|  | **MSSA** (n=160) | | **MRSA** (n=87) | | | χ2 or T-tests p-value | | **MSSA** (n=111) | | **MRSA** (n=46) | | χ2 or T-test |
|  | Mean (%) | 95%CI | Mean (%) | 95%CI | |  |  | Mean (%) | 95%CI | Mean (%) | 95%CI | p-value |
| **Outcome variables** |  |  |  |  | |  |  | |  |  |  |  |
| Overall mortality (%) | 24.38% | 18—32 | 34.48% | 25—45 | | 0.092 | 33.33% | | 25—43 | 45.65% | 31—61 | 0.147 |
| Full hospital LOS (days) | 31.38 | 6—100 | 42.23 | 5—111 | | 0.020 | 35.32 | | 6—129 | 48.56 | 5—120 | 0.082 |
| LOS before BC (days) | 7.83 | 0—28 | 15.56 | 0—53 | | <0.001 | 9.45 | | 0—34 | 22.09 | 0—87 | <0.001 |
| LOS after BC (days) | 24.85 | 1—82 | 29.83 | 2—72 | | 0.272 | 28.12 | | 1—110 | 27.30 | 1—119 | 0.914 |
| Full ICU admission (%) | 38.75% | 31—47 | 44.83% | 34—56 | | 0.355 | 41.44% | | 32—51 | 50.00% | 35—65 | 0.329 |
| ICU admission (%) before BC | 7.50% | 4—13 | 2.30% | 0—8 | | 0.092 | 7.21% | | 3—14 | 4.35% | 1—15 | 0.507 |
| ICU admission (%) after BC | 26.88% | 20—34 | 37.93% | 28—49 | | 0.073 | 31.53% | | 23—41 | 45.65% | 31—61 | 0.094 |
| Full ICU LOS (days) | 5.22 | 0—23 | 12.18 | 0—60 | | 0.001 | 8.38 | | 0—39 | 12.91 | 0—57 | 0.146 |
| ICU LOS after BC (days) | 4.49 | 0—22 | 11.94 | 0—60 | | 0.001 | 7.30 | | 0—39 | 11.83 | 0—57 | 0.137 |
| **Independent variables** |  |  |  |  | |  |  | |  |  |  |  |
| Age (years) | 58.65 | 26—81 | 60.48 | 36—82 | | 0.370 | 61.81 | | 37—86 | 58.79 | 35—84 | 0.264 |
| Hospitalisation in last three months (%) | 35.03% | 28—43 | 38.96% | 28—51 | | 0.559 | 30.56% | | 22—40 | 18.60% | 8—33 | 0.138 |
| Antibiotic consumption in last three months (%) | 18.94% | 13—27 | 32.89% | 23—45 | | 0.023 | 20.00% | | 12—29 | 16.28% | 7—31 | 0.608 |
| CCI (mean) | 3.39 | 0—800 | 2.89 | 0—8 | | 0.152 | 3.92 | | 0—9 | 3.26 | 0—9 | 0.166 |
| Null, CCI = 0 (%) | 15.00% | 10—21 | 18.39% | 11—28 | | 0.492 | 9.91% | | 5—17 | 23.91% | 13—39 | 0.021 |
| Mild, CCI = 1 or 2 (%) | 31.25% | 24—39 | 28.74% | 20—39 | | 0.683 | 24.32% | | 17—33 | 26.09% | 14—41 | 0.818 |
| Moderate, CCI = 3 or 4 (%) | 21.88% | 16—29 | 29.89% | 21—41 | | 0.165 | 24.32% | | 17—33 | 17.39% | 8—31 | 0.345 |
| Severe, CCI ≥ 5 (%) | 31.88% | 25—40 | 22.99% | 15—33 | | 0.142 | 41.44% | | 32—51 | 32.61% | 20—48 | 0.305 |
| Source of the BSI |  |  |  |  | |  |  | |  |  |  |  |
| Primary (%) | 28.28% | 20—38 | 31.25% | 21—43 | | 0.668 | 31.43% | | 21—44 | 41.46% | 26—58 | 0.289 |
| Catheter (%) | 29.29% | 21—39 | 8.75% | 4—17 | | 0.001 | 14.29% | | 7—25 | 17.07% | 7—32 | 0.697 |
| Pneumonia/respiratory (%) | 24.24% | 16—34 | 21.25% | 13—32 | | 0.638 | 22.86% | | 14—34 | 24.39% | 12—40 | 0.856 |
| Gastrointestinal (%) | 4.04% | 1—10 | 11.25% | 5—20 | | 0.065 | 2.86% | | 0—10 | 4.88% | 1—17 | 0.585 |
| Abdomen (%) | 8.08% | 4—15 | 11.25% | 5—20 | | 0.475 | 10.00% | | 4—20 | 12.20% | 4—26 | 0.722 |
| Bones and joints (%) | 3.03% | 1—9 | 7.50% | 3—16 | | 0.176 | 7.14% | | 2—16 | 0.00% | 0—9 | 0.081 |
| Skin and soft tissue (%) | 3.03% | 1—9 | 8.75% | 4—17 | | 0.099 | 10.00% | | 4—20 | 0.00% | 0—9 | 0.037 |
| Meningitis (%) | 0.00% | 0—4 | 0.00% | 0—5 | | 0.999 | 1.43% | | 0—8 | 0.00% | 0—9 | 0.447 |
| Community-acquired infection (%) | 28.21% | 21—36 | 23.81% | 15—34 | | 0.465 | 33.33% | | 25—43 | 20.93% | 10—36 | 0.134 |
| Indwelling catheter (%) | 25.63% | 19—33 | 43.68% | 33—55 | | 0.004 | 27.03% | | 19—36 | 54.35% | 39—69 | 0.001 |
| Kidney therapy before BC (%) | 24.80% | 18—33 | 5.13% | 1—13 | | <0.001 | 17.28% | | 10—27 | 9.76% | 3—23 | 0.272 |
| Transfer from another hospital (%) | 23.75% | 17—31 | 12.94% | 7—22 | | 0.044 | 21.62% | | 14—30 | 8.89% | 2—21 | 0.061 |
| ID specialist consultation (%) | 40.54% | 29—53 | 49.38% | 38—61 | | 0.272 | 38.33% | | 26—52 | 58.97% | 42—74 | 0.045 |
| Mechanical ventilation before BC (%) | 6.25% | 3—11 | 3.45% | 1—10 | | 0.348 | 2.70% | | 1—8 | 6.52% | 1—18 | 0.259 |
| Mechanical ventilation after BC (%) | 10.00% | 6—16 | 40.23% | 30—51 | | <0.001 | 23.42% | | 16—32 | 45.65% | 31—61 | 0.005 |
| Surgery previous BC (%) | 0.63% | 0—3 | 1.15% | 0—6 | | 0.662 | 0.90% | | 0—5 | 0.00% | 0—8 | 0.522 |
| Surgery after BC (%) | 5.63% | 3—10 | 18.39% | 11—28 | | 0.001 | 2.70% | | 1—8 | 10.87% | 4—24 | 0.034 |
| **Antibiotic consumption in daily defined doses ‘DDDs’ per treatment course after BC** | | | | |  |  |  |  |  |  |  |  |
| Total consumption (DDDs per 1,000 bed-days) | 151.73 | 0—612 | 317.55 | 0—947 | | <0.001 | 128.38 | | 0—112 | 255.68 | 0—164 | 0.001 |
| Carbapenems (DDDs per 1,000 bed-days) | 6.12 | 0—44 | 30.89 | 0—124 | | <0.001 | 5.33 | | 0—28 | 19.41 | 0—82 | 0.001 |
| Cephalosporins (DDDs per 1,000 bed-days) | 48.10 | 0—262 | 73.31 | 0—206 | | 0.020 | 39.47 | | 0—169 | 66.30 | 0—137 | 0.033 |
| Macrolides (DDDs per 1,000 bed-days) | 1.31 | 0—0 | 13.18 | 0—82 | | <0.001 | 0.71 | | 0—0 | 9.89 | 0—55 | 0.001 |
| Fluoroquinolones (DDDs per 1,000 bed-days) | 3.94 | 0—0 | 15.24 | 0—82 | | <0.001 | 4.98 | | 0—56 | 2.93 | 0—0 | 0.516 |
| Aminoglycoside (DDDs per 1,000 bed-days) | 6.56 | 0—44 | 27.59 | 0—206 | | <0.001 | 2.49 | | 0—0 | 11.72 | 0—55 | 0.014 |
| Tetracyclines (DDDs per 1,000 bed-days) | 0.00 | 0—0 | 1.24 | 0—0 | | 0.176 | 0.00 | | 0—0 | 1.10 | 0—0 | 0.121 |
| Penicillin (DDDs per 1,000 bed-days) | 47.66 | 0—306 | 27.59 | 0—165 | | 0.128 | 45.52 | | 0—197 | 19.78 | 0—82 | 0.107 |
| Glycopeptides (DDDs per 1,000 bed-days) | 28.42 | 0—131 | 81.96 | 0—288 | | <0.001 | 21.34 | | 0—84 | 93.41 | 0—246 | <0.001 |
| LOT (days) | 9.93 | 0—42 | 34.56 | 0—95 | | <0.001 | 11.23 | | 0—37 | 32.48 | 0—90 | <0.001 |
| NOA (number) | 1.65 | 0—7 | 4.92 | 0—13 | | <0.001 | 1.50 | | 0—5 | 4.59 | 0—16 | <0.001 |

Notes: ARB= Antibiotic resistance. ASB= Antibiotic sensitive. BSI= Bloodstream infection. LOT= length of therapy defined as number of days a patient receives any antibiotic. NOA= Number of antibiotics used for treating a patient. CCI= Charlson comorbidity index. 95% CI for proportion variables were estimated49. BC= index Blood culture. ID= Infectious disease. ICU= Intensive care unit. LOS= Length of hospital stay. bχ2 or T-test were employed according to each variable’s distribution (α=0.05).

# V. Supplementary results: propensity score and inverse probability weights

The propensity score is estimated to balance observable covariates between treatment groups in observational studies, allowing us to approximate the conditions of a randomized trial. Specifically, when investigating the prevalence of CRE or MRSA a logistic regression model is employed (separately for each pathogen) where the treatment assignment (e.g., exposure to a specific antibiotic) is regressed on various confounding factors such as age, prior hospitalisation, and other relevant clinical variables. This model computes the probability (propensity score) for everyone receiving the treatment, given their covariates.

Once propensity scores are estimated, they are utilised in Inverse Probability Weighting (IPW) to adjust for differences in baseline characteristics between treatment groups. The weights are calculated as follows:

For individuals who received the treatment (treated group; either CRE or MRSA):

Weight_1_ =1 / propensity score

For individuals who did not receive the treatment (control group; either CSE or MSSA):

Weight_2_= 1/ (1-propensity score)

These weights help create a synthetic sample where the distribution of covariates is similar across both treated and untreated groups, mitigating the effects of confounding. The weighted sample is then used in subsequent statistical analyses to estimate the effect of treatment (CRE/MRSA) on outcomes (ICU Admission, mortality, LOS) ensuring that the estimates more accurately reflect what would be observed in a randomized controlled experiment.

**Table A8.** Results of the propensity score matching estimation (PSM) using a logistic model among patients with carbapenem-resistant Enterobacterales (CRE) bloodstream infections (BSI)

| **Variable** | **OR** | **95% CI** | **p-value** |
| --- | --- | --- | --- |
| Age in years | 0.98 | 0.96—1.01 | 0.047 |
| Sex (Ref. male) | 0.44 | 0.28—0.70 | 0.001 |
| Previous hospitalisation | 0.95 | 0.53—1.73 | 0.876 |
| Use of a catheter previous BC | 3.22 | 1.86—5.59 | 0.003 |
| Kidney therapy before BC (%) | 0.57 | 0.18—1.78 | 0.332 |
| Hospital LOS before BC, logged | 1.01 | 0.99—1.02 | 0.235 |
| Community-acquired infection | 0.49 | 0.26—0.90 | 0.022 |
| CCI | 1.03 | 0.93—1.14 | 0.591 |
| ICU admission before BC | 1.09 | 1.02—1.43 | 0.002 |
| Mechanical ventilation before BC | 1.60 | 0.48—5.33 | 0.444 |
| Year (Reference: 2018/2019) |  |  |  |
| 2020 | 2.49 | 1.26—4.89 | 0.008 |
| 2021 | 1.27 | 0.67—2.39 | 0.465 |
| 2022 | 0.47 | 0.15—1.51 | 0.207 |
| Name of the hospital (Reference: Hospital 1) | | | |
| Hospital 2 | 42.93 | 17.31—106.44 | <0.001 |
| Hospital 3 | 23.83 | 9.49—59.84 | <0.001 |
| Constant term | 0.11 | 0.03—0.42 | 0.001 |

Notes: CCI= Charlson Comorbidity Index. LOS= Length of hospital stay. ICU= Intensive care unit. OR= Odds ratio. CI= Confidence interval. BC= index blood culture. BSI= Bloodstream infection. Table S6·1·1 displays no statistically significant differences prior to index BSI culture concerning sociodemographic and underlying health characteristics after employing IPW-adjusted statistics. The large odd ratios reflect that one hospital had little CRE (8/79, 10%), while the second and third hospitals had higher CRE prevalence (124/182,68%, and 101/208, 49%, respectively).


**Figure A3.** Density of the propensity score estimated by CRE/CSE group, hospital patients. CRE= Carbapenem-resistant Enterobacterales. CSE= Carbapenem-susceptible Enterobacterales.

**Table A9.** Results of the propensity score matching estimation (PSM) using a logistic model among patients with methicillin-resistant *Staphylococcus aureus* (MRSA) bloodstream infections (BSI)

| **Variable** | **OR** | **95% CI** | **p-value** |
| --- | --- | --- | --- |
| Age in years | 1.02 | 1.00—1.04 | 0.104 |
| Sex (Ref. male) | 0.73 | 0.48—1.01 | 0.050 |
| Previous hospitalisation | 2.18 | 1.14—4.18 | 0.019 |
| Use of a catheter previous BC | 1.71 | 0.95—3.09 | 0.073 |
| Kidney therapy before BC (%) | 0.50 | 0.19—1.28 | 0.148 |
| Hospital LOS before BC, logged | 1.04 | 1.02—1.05 | <0.001 |
| Community-acquired infection | 1.42 | 0.71—2.85 | 0.317 |
| CCI | 0.87 | 0.76—1.01 | 0.073 |
| ICU admission before BC | 0.95 | 0.23—3.92 | 0.941 |
| Mechanical ventilation before BC | 1.15 | 0.33—4.05 | 0.831 |
| Year (Reference: 2018/2019) |  |  |  |
| 2020 | 2.6 | 1.17—5.77 | 0.019 |
| 2021 | 1.53 | 0.71—3.31 | 0.277 |
| 2022 | 0.83 | 0.24—2.82 | 0.764 |
| Name of the hospital (Reference: Hospital 1) | | | |
| Hospital 2 | 0.98 | 0.49—1.94 | 0.946 |
| Hospital 3 | 0.12 | 0.06—0.23 | <0.001 |
| Constant term | 0.17 | 0.04—0.75 | 0.019 |

Notes: CCI= Charlson Commorbidity Index. LOS= Length of hospital stay. ICU= Intensive care unit. OR= Odds ratio. CI= Confidence interval. BC= index blood culture. BSI= Bloodstream infection. *Age in years was mean centred and subtracted by its standard deviation. Table S6·1·1 displays no statistically significant differences prior to index BSI culture concerning sociodemographic and underlying health characteristics after employing IPW-adjusted statistics.

 **Figure A4.** Density of the propensity score estimated by MRSA/MSSA group, hospital patients. Notes: MRSA= Methicillin-resistant Staphylococcus aureus. MSSA= Methicillin-susceptible *Staphylococcus aureu*s.

# VI. Supplementary methods: regression and survival analyses

First, we computed pathogen-specific (either for CRE/MRSA) IPW-adjusted logistic regressions for the ICU admission outcome. We used Sex at birth as the only control variable. Marginal effects were computed for sex-specific estimates and their relationship with our outcome variable (ICU admission). We evaluated multicolinearity and overall was VIF<10 in our models. We clustered standard errors by hospital site.

Second, we employed survival regression analyses using COX-models and accounting for competing-risks (i.e., mortality and discharge)^58^ to determine the co-hazards associated with sex, ARB (e.g., MRSA/MSSA or CRE/CSE) and disease severity (e.g., general wards or ICU admission). To do so, we employed three-way interaction terms between sex, ARB, and disease severity. Time was added as number of days in the hospital, since disease diagnosis (BSI) and up until discharge or death. We focused on in-hospital mortality only, and our COX model with competing risks were weighted using previously estimated IPW. Subsequently, we predicted the mortality values and plotted the cumulative incidence of mortality on time (in days) and across all our different groups derived from interaction terms. These 8 groups were; (1) ARB ✱ Male ✱ GW, (2) ARB ✱ Male ✱ GW, (3) ARB ✱ Female ✱ GW, (4) ARB ✱ Female ✱ GW, (5) ARB ✱ Male ✱ICU, (6) ARB ✱ Male ✱ ICU, (7) ARB ✱ Female ✱ ICU, and (8) ARB ✱ Female ✱ ICU. ARB depended upon model description, either CRE or MRSA. Multicolinearity was tested and overall was VIF<10. We clustered standard errors by hospital site.

# VII. Supplementary results: Inverse-probability weighted regression results

**Table A10.** Inverse-probability weighting-adjusted regression results of the effect of antibiotic-resistant Enterobacterales and *Staphylococcus aureus* on ICU admission.

| **(A) Overall** | | | |
| --- | --- | --- | --- |
| **I. Enterobacterales** | | | |
| **Outcome (ICU admission)** | **OR** | **95% CI** | **p-value** |
| Carbapenem-resistant Enterobacterales (CRE) | 1.37 | 1.04—1.8 | 0.024 |
| Sex (Ref.: Women/females) | 0.88 | 0.67—1.16 | 0.381 |
| Constant term | 0.62 | 0.50-0.77 | <0.001 |
| **II. *Staphylococcus aureus*** | | | |
| **Outcome (ICU admission)** | **OR** | **95% CI** | **p-value** |
| Methicilin-resistant *Staphylococcus aureus* (MRSA) | 1.38 | 1.00—1.81 | 0.048 |
| Sex (Ref.: Men/males) | 1.33 | 0.99—1.73 | 0.050 |
| Constant term | 0.39 | 0.31-0.49 | <0.001 |

Notes: ICU= Intensive care unit. OR= Odds ratio. IRR= Incidence risk ratio. CI= Confidence interval. p= p-value. HR= Hazard ratio. Const.= Constant term.

**
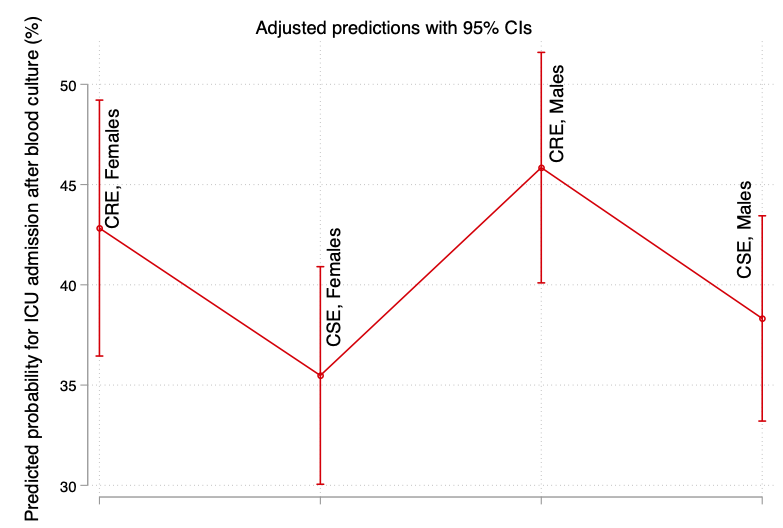
**

**Figure A5** Sex-specific marginal effects for ICU admission after blood culture index among patients with CRE or CSE bloodstream infections. Functions were computed from the IPW-adjusted logistic model. CRE= Carbapenem-resistant Enterobacterales. CSE= Carbapenem-susceptible Enterobacterales. Predicted probabilities were CRE Females (42.83%, 95%CI=36.45-49.21%), CSE Females (35.48%, 95%CI=30.05-40.91%), CRE Males (45.85%, 95%CI=40.09-51.59%), CSE Males (38.32%, 95%CI=33.2-43.44%).


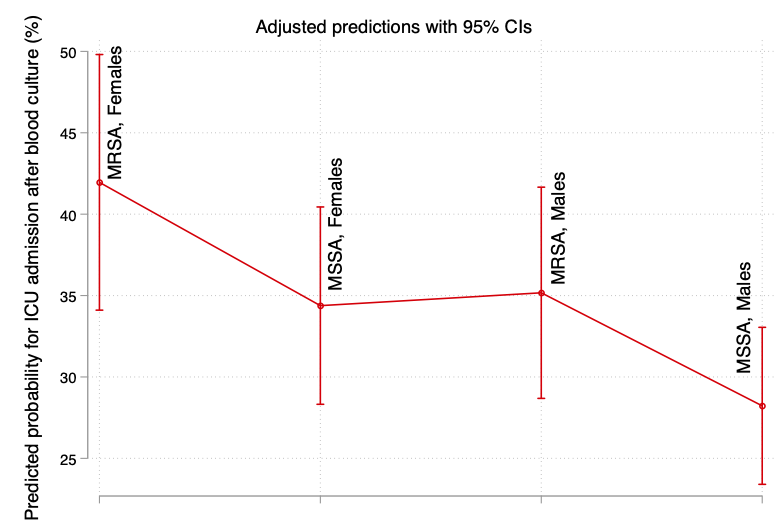


**Figure A6** Sex-specific marginal effects for ICU admission after blood culture index among patients with MRSA or MSSA bloodstream infections. Functions were computed from the IPW-adjusted logistic model. MRSA= Methicillin-resistant Staphylococcus aureus. MSSA= Methicillin-susceptible Staphylococcus aureus. Predicted probabilities were MRSA Females (41.96%, 95%CI=34.10-49.81%), MSSA Females (34.38%, 95%CI=28.68-41.66%), MRSA Males (35.17%, 95%CI= 28.68-41.66%), MSSA Males (28.26%, 95%CI= 23.40-33.05%).

**Table A11.** Inverse-probability weighting-adjusted survival regression results with competing risks to explore the effect of antibiotic-resistant Enterobacterales and *Staphylococcus aureus* on mortality.

| **I. Carbapenem-resistant Enterobacterales (CRE)** | | | |
| --- | --- | --- | --- |
| **Outcome** | **HR** | **95% CI** | **p-value** |
| CSE ✱ Male ✱ GW (Reference, mortality proportion=22.8%) |  |  |  |
| CRE ✱ Male ✱ GW | 1.80 | 1.2—2.8 | 0.010 |
| CSE ✱ Female ✱ GW | 0.81 | 0.5—1.3 | 0.396 |
| CRE ✱ Female ✱ GW | 0.55 | 0.3—1.0 | 0.062 |
| CSE ✱ Male ✱ICU | 1.62 | 1.0—2.6 | 0.036 |
| CRE ✱ Male ✱ ICU | 1.30 | 0.8—2.1 | 0.270 |
| CSE ✱ Female ✱ ICU | 2.23 | 1.4—3.6 | <0.001 |
| CRE ✱ Female ✱ ICU | 2.40 | 1.5—3.9 | <0.001 |
| **II. Methicillin-resistant  *Staphylococcus aureus* (MRSA)** | | | |
| **Outcome** | **HR** | **95% CI** | **p-value** |
| MSSA ✱ Male ✱ GW (Reference, mortality proportion=23.1%) |  |  |  |
| MRSA ✱ Male ✱ GW | 1.01 | 0.6—1.7 | 0.960 |
| MSSA ✱ Female ✱ GW | 1.22 | 0.8—1.9 | 0.374 |
| MRSA ✱ Female ✱ GW | 2.25 | 1.3—3.8 | 0.002 |
| MSSA ✱ Male ✱ICU | 1.10 | 0.6—1.9 | 0.719 |
| MRSA ✱ Male ✱ ICU | 2.32 | 1.5—3.7 | <0.001 |
| MSSA ✱ Female ✱ ICU | 2.07 | 1.3—3.3 | 0.002 |
| MRSA ✱ Female ✱ ICU | 2.34 | 1.3—4.1 | 0.003 |

Notes: ICU= Intensive care unit. LOS= Length of hospital stay. OR= Odds ratio. IRR= Incidence risk ratio. CI= Confidence interval. p= p-value. HR= Hazard ratio. Const.= Constant term. We applied VIF test (Variance-inflator factor) and VIF=1.14, showing no multicollinearity. All possible interactions terms were tested between independent variables and utilising the Wald test; only those statistically significant at 10% level were included. GW= General ward. MSSA= Methicillin-susceptible *Staphylococcus aureus*. MRSA= Methicillin-resistant *Staphylococcus aureus*. CSE= Carbapenem-susceptible Enterobacterales. CRE= Carbapenem-resistant Enterobacterales.


Figure A7. Cumulative incidence of mortality utilising an inverse-probability weighting-adjusted survival model with competing risks to explore the effects of methicillin-resistant Staphylococcus aureus on mortality, across gender and hospital ward specifications**.** MRSA= Methicillin-resistant *Staphylococcus aureus*. MSSA= Methicillin-susceptible *Staphylococcus aureus*. ICU= Intensive care unit. GW= General ward.

**Figure A8.** Cumulative incidence of mortality utilising an inverse-probability weighting-adjusted survival model with competing risks to explore the effects of carbapenem-resistant Enterobacterales on mortality, across gender and hospital ward specifications. CRE= Carbapenem-resistant Enterobacterales. CSE= Carbapenem-susceptible Enterobacterales. ICU= Intensive care unit. GW= General ward.

**Table A12.** Prior hospital stay among hospital-acquired infections, by sex and resistant pathogen.

| **Pathogen** | **Sex** | **Hospital stays [days, mean]** | **Standard deviation** | **Number of observations** |
| --- | --- | --- | --- | --- |
| MRSA | Men | 21.08 | 22.93 | 63 |
| MRSA | Women | 29.44 | 26.07 | 32 |
| MSSA | Men | 10.94 | 11.79 | 108 |
| MSSA | Women | 14.24 | 18.95 | 71 |
| CRE | Men | 21.86 | 18.57 | 129 |
| CRE | Women | 26.53 | 18.87 | 62 |
| CSE | Men | 20.04 | 20.75 | 79 |
| CSE | Women | 16.66 | 11.85 | 62 |

Notes: CRE= Carbapenem-resistant Enterobacterales. CSE= Carbapenem-susceptible Enterobacterales. ICU= Intensive care unit. GW= General ward. MRSA= Methicillin-resistant *Staphylococcus aureus*. MSSA= Methicillin-susceptible *Staphylococcus aureus*

**Table A13**. Hospital stays after infection diagnostic, by sex, ICU admission, resistant pathogens and mortality or recovery status.

| **Pathogen** | **Sex** | **ICU or GW** | **Dead or recovered** | **Median LOS conditional on subpopulations** |
| --- | --- | --- | --- | --- |
| MRSA | Men | ICU | Dead | 20 |
| MRSA | Men | ICU | Recovered | 23 |
| MRSA | Women | ICU | Dead | 13 |
| MRSA | Women | ICU | Recovered | 26 |
| MRSA | Men | GW | Dead | 11 |
| MRSA | Men | GW | Recovered | 18 |
| MRSA | Women | GW | Dead | 14 |
| MRSA | Women | GW | Recovered | 19 |
|  |  |  |  |  |
| MSSA | Men | ICU | Dead | 12 |
| MSSA | Men | ICU | Recovered | 11 |
| MSSA | Women | ICU | Dead | 20 |
| MSSA | Women | ICU | Recovered | 16 |
| MSSA | Men | GW | Dead | 11 |
| MSSA | Men | GW | Recovered | 19 |
| MSSA | Women | GW | Dead | 14 |
| MSSA | Women | GW | Recovered | 17 |
|  |  |  |  |  |
| CRE | Men | ICU | Dead | 21 |
| CRE | Men | ICU | Recovered | 26 |
| CRE | Women | ICU | Dead | 31 |
| CRE | Women | ICU | Recovered | 30 |
| CRE | Men | GW | Dead | 7 |
| CRE | Men | GW | Recovered | 20 |
| CRE | Women | GW | Dead | 20 |
| CRE | Women | GW | Recovered | 23 |
|  |  |  |  |  |
| CSE | Men | ICU | Dead | 12 |
| CSE | Men | ICU | Recovered | 20 |
| CSE | Women | ICU | Dead | 10 |
| CSE | Women | ICU | Recovered | 18 |
| CSE | Men | GW | Dead | 11 |
| CSE | Men | GW | Recovered | 14 |
| CSE | Women | GW | Dead | 9 |
| CSE | Women | GW | Recovered | 15 |

Notes: CRE= Carbapenem-resistant Enterobacterales. CSE= Carbapenem-susceptible Enterobacterales. ICU= Intensive care unit. GW= General ward. MRSA= Methicillin-resistant *Staphylococcus aureus*. MSSA= Methicillin-susceptible *Staphylococcus aureus*. LOS= Length of hospital stay in days.

# VIII. Mathematical model results


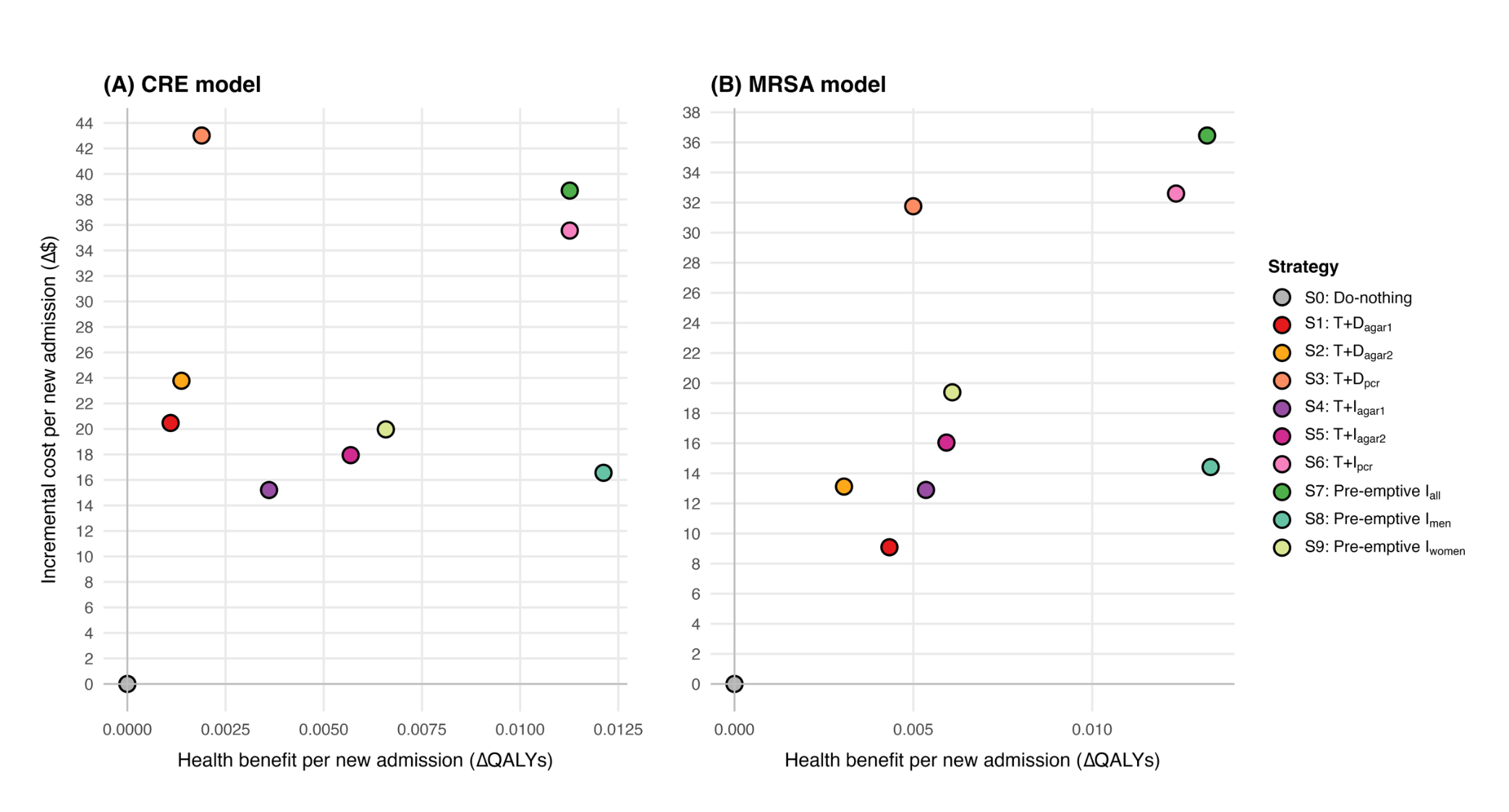


**Figure A9**. Incremental costs and health benefits per new admission, by strategy and pathogen model. CRE= Carbapenem-resistant Enterobacterales. MRSA= Methicillin-resistant *Staphylococcus aureus.* QALYs= Quality-adjusted life years. Costs were measured in USD.

**
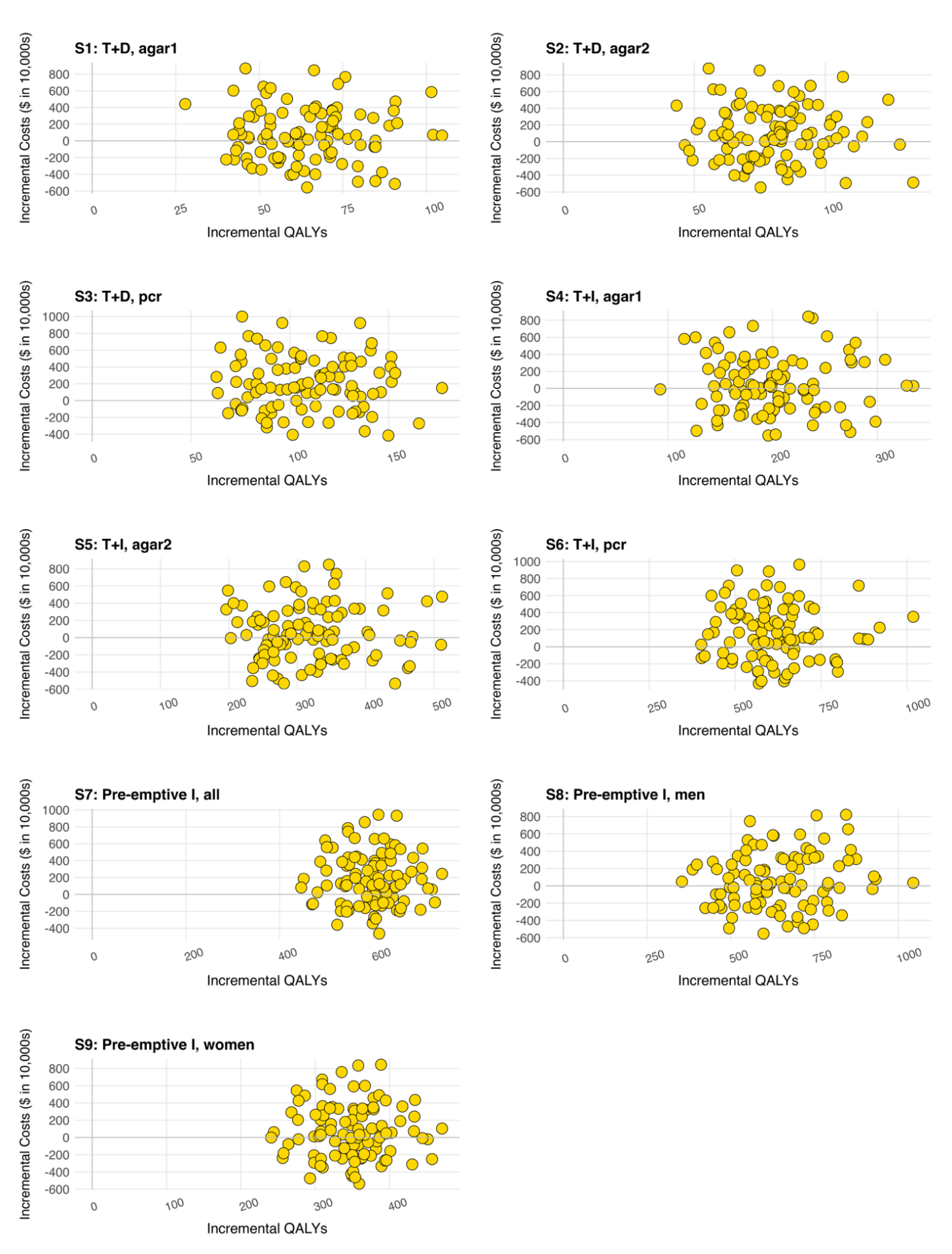
**

**Figure A10.** Probability sensitivity analysis for incremental costs and QALYs across strategies to reduce CRE transmission. QALYs= Quality-adjusted life years. T+D= Test + decolonisation treatment. T+I= Test + isolation. I= isolation. 1,000 observations were grouped into 100 for visualisation purposes.

**
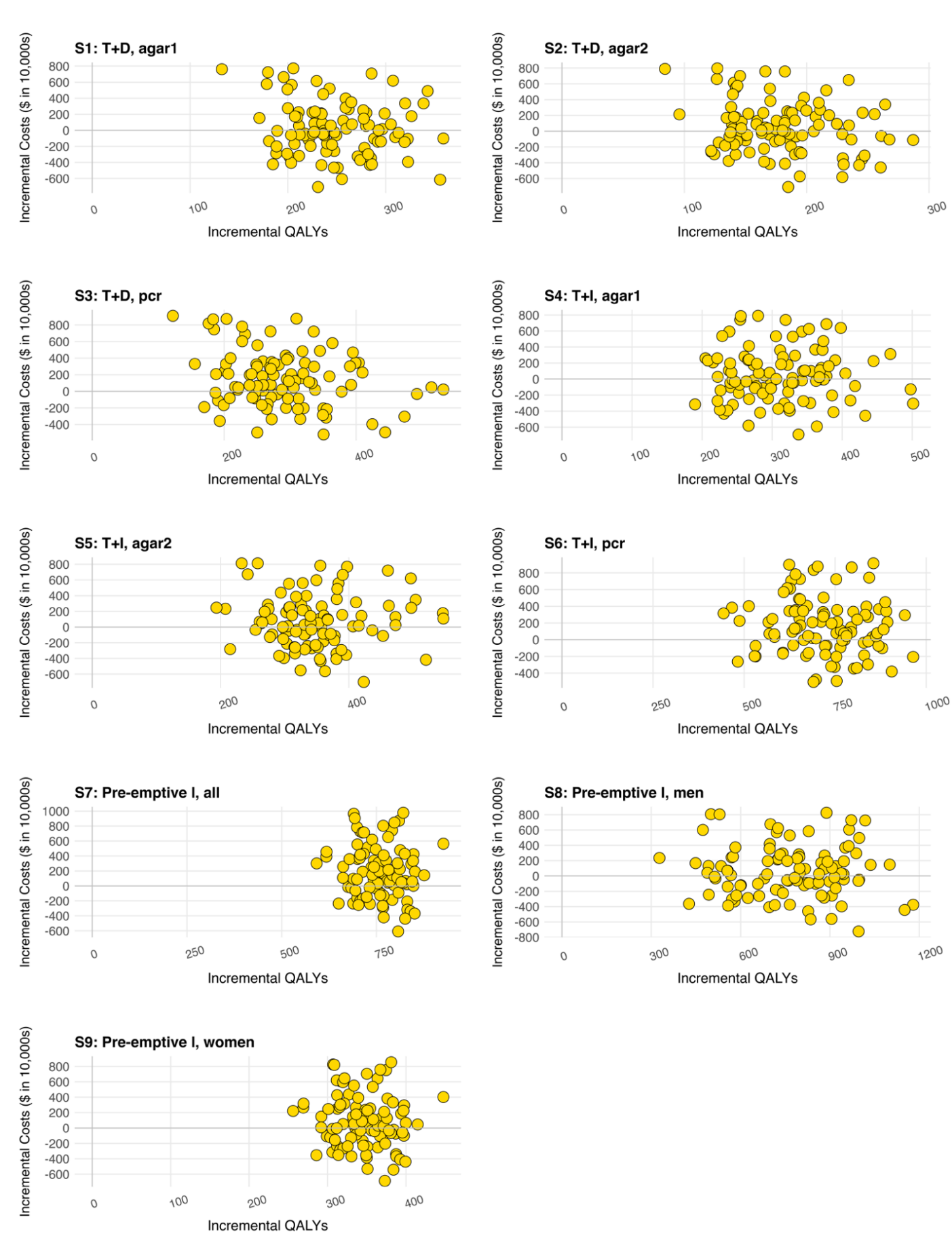
 Figure A11.** Probability sensitivity analysis for incremental costs and QALYs across strategies to reduce MRSA transmission. QALYs= Quality-adjusted life years. T+D= Test + decolonisation treatment. T+I= Test + isolation. I= isolation. 1,000 observations were grouped into 100 for visualisation purposes.

**
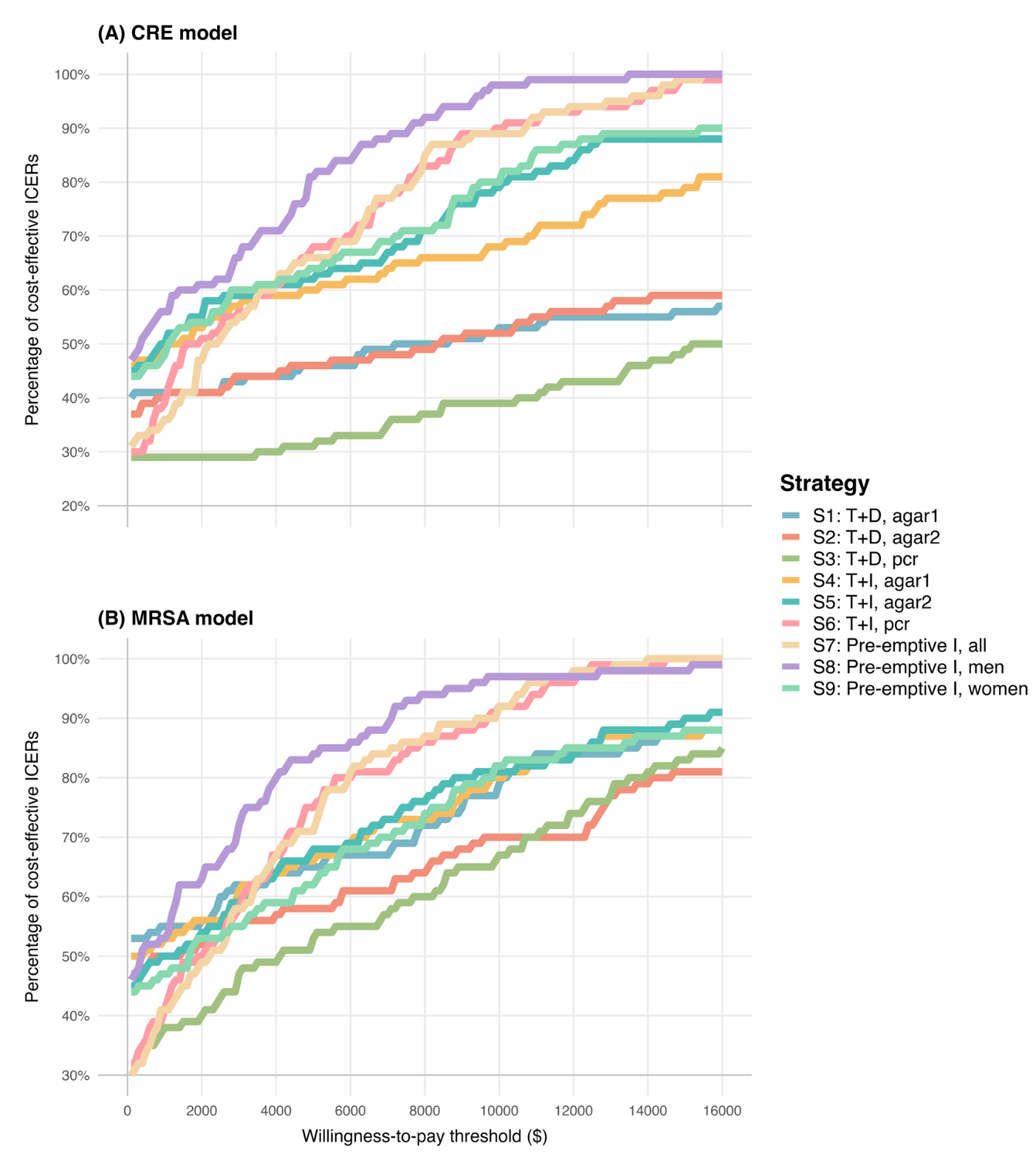
**

**Figure A12.** Probability sensitivity analyses results and percentage of simulations indicating cost-effectiveness by intervention and willingness-to-pay thresholds. ICER= Incremental cost-effectiveness ratio. Chile’s Gross Domestic Product (GDP) per capita is $15,356 approximately. T+D= testing + decolonization treatment. T+I= Testing + isolation (contact precaution). Pre-emptive I= Pre-emptive isolation (contact precaution).

**
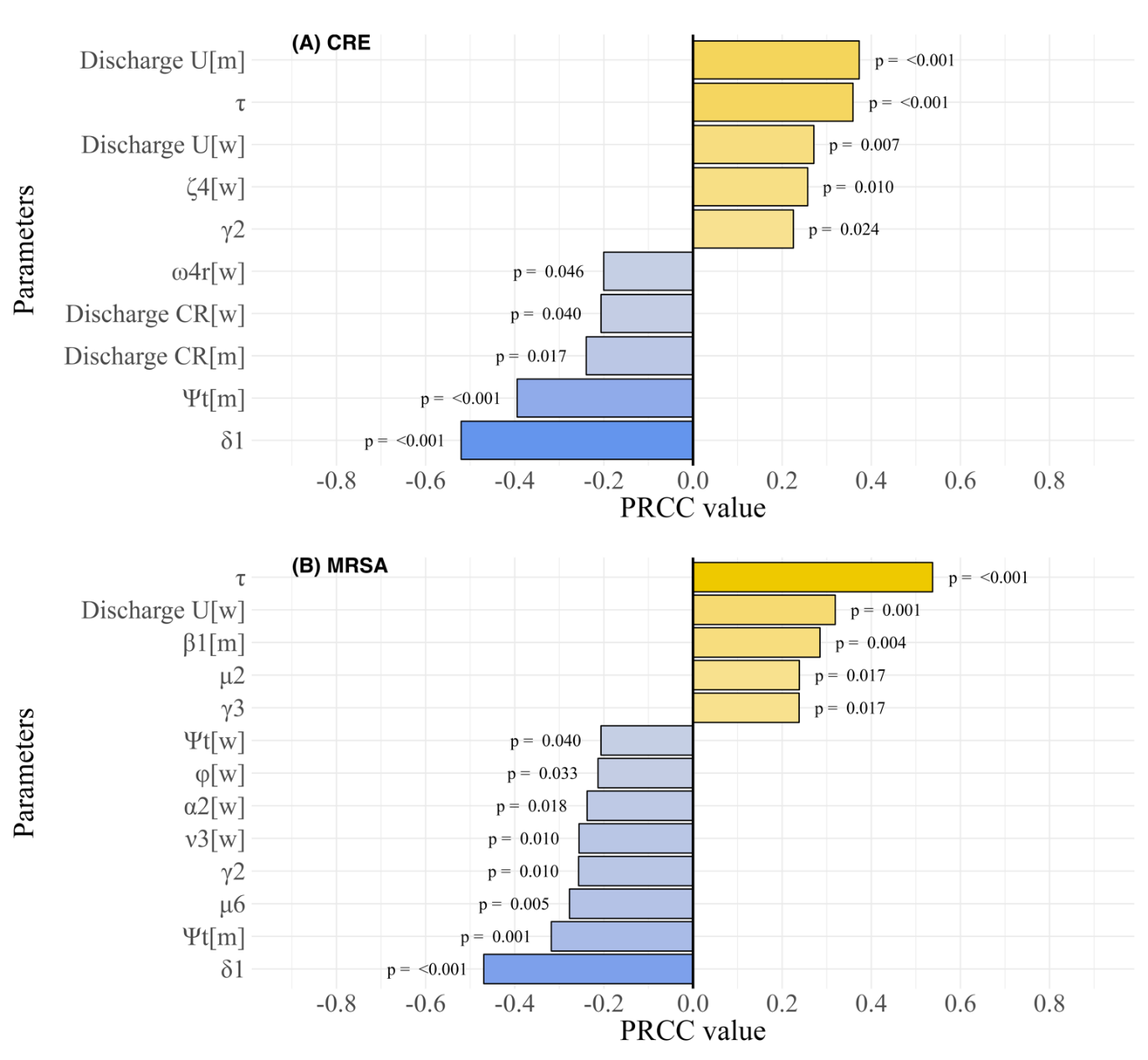
**

**Figure A13.** Most sensible parameters from the mathematical model, by pathogen. Parameters reporting a p-value<0.05 were included because they are influential regarding the total number of colonized and infected individuals. Full list of parameters’ PRCC values are shown in Supplementary Figures A2. We used the Latin hypercube sampling to compute the partial rank correlation (PRCC) values. Negative PRCC means a negative relationship between model input and number of total individuals colonized/infected by CRE/MRSA; and values>0 indicate a positive relationship between the parameter and the total number of individuals colonized/infected by CRE/MRSA. Yellow values are for positive correlations and blue colours for negatives. CRE= Carbapenem-resistant Enterobacterales. MRSA= Methicillin-resistant *Staphylococcus aureus*.


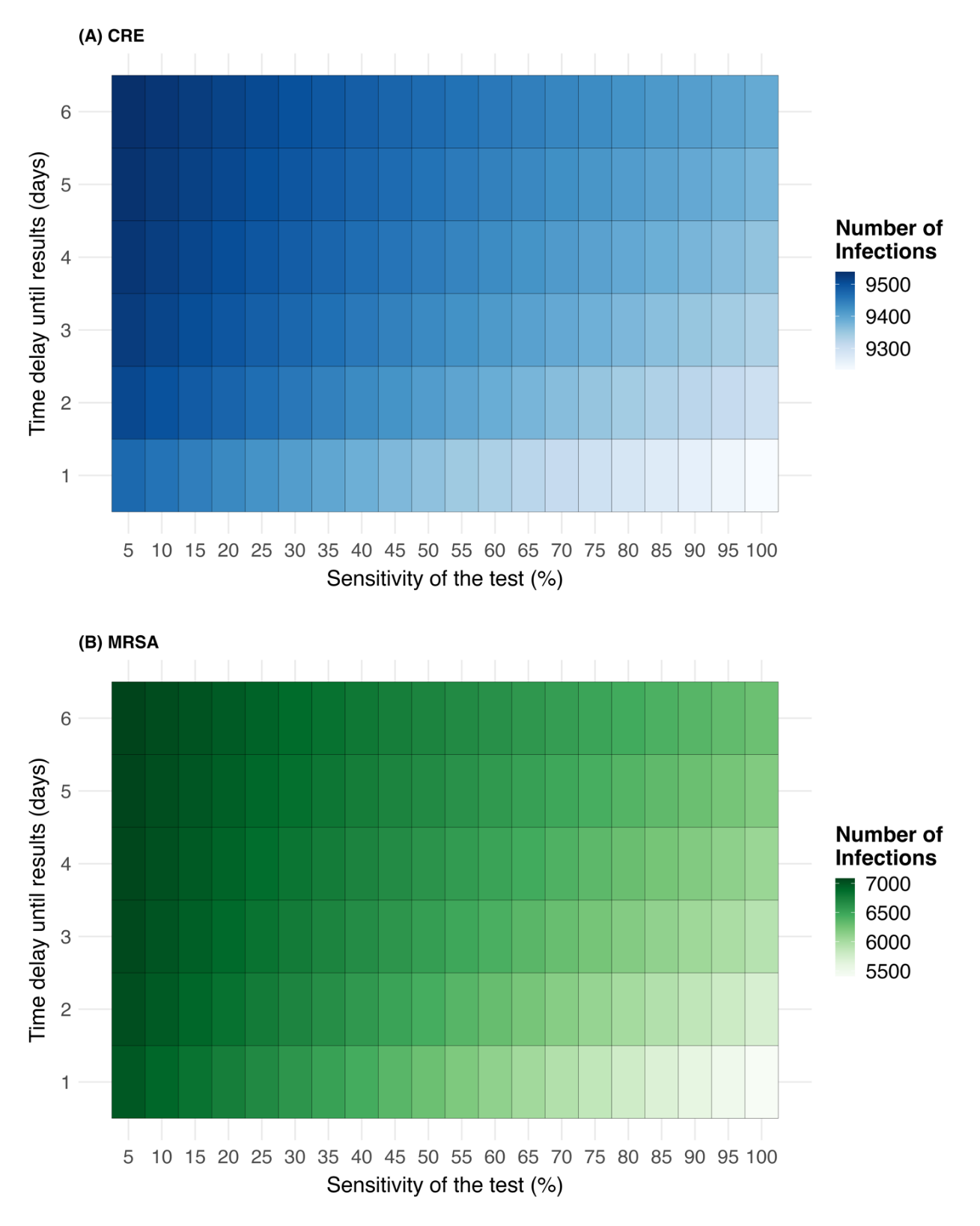


**Figure A14.** Dual impact of test sensitivity and time delay for test results on the number of infections overtime if individuals are tested and decolonised, by pathogen. CRE= Carbapenem-resistant Enterobacterales. MRSA= Methicillin-resistant *Staphylococcus aureus*.


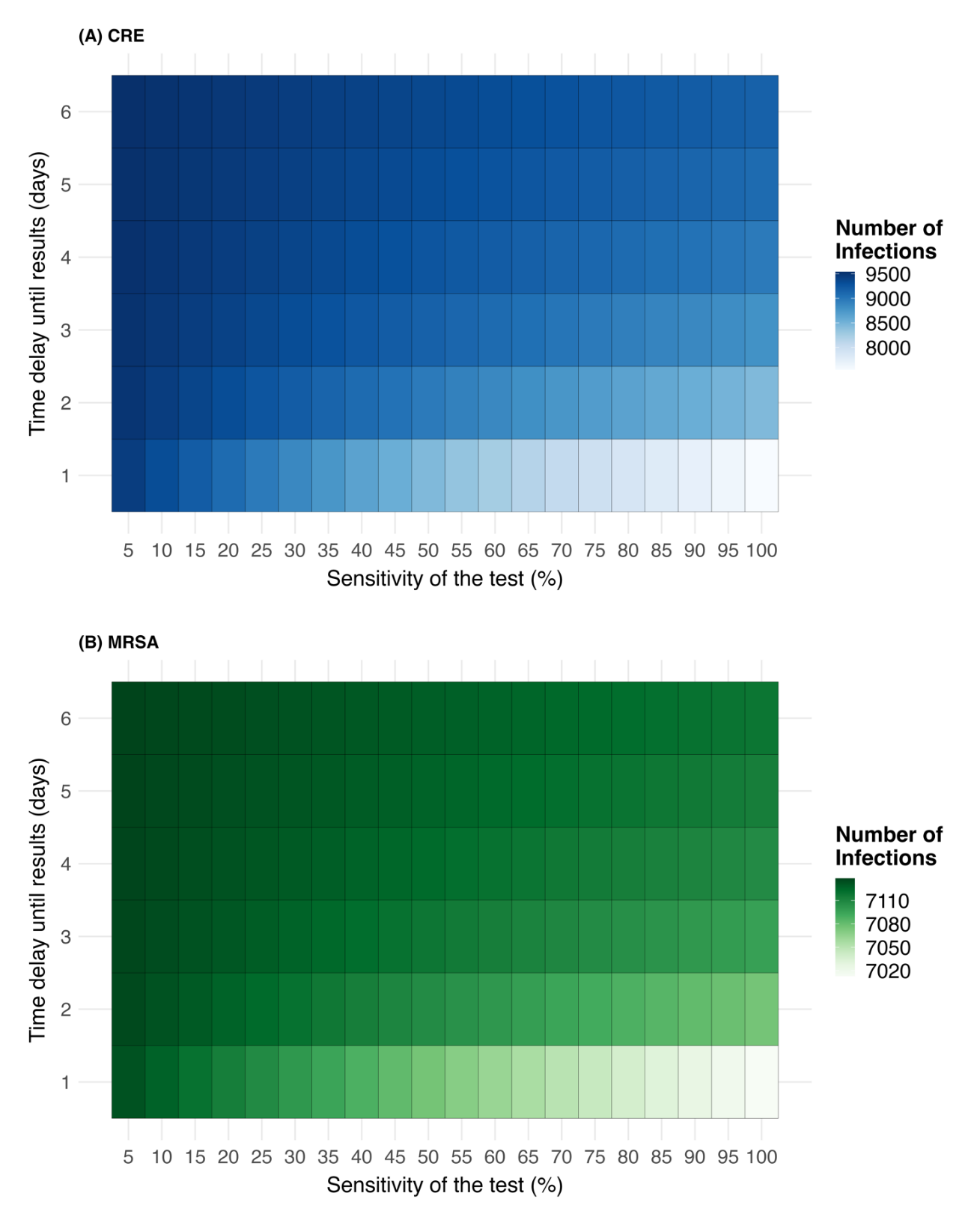


**Figure A15.** Dual impact of test sensitivity and time delay for test results on the number of infections overtime if individuals are tested and isolated (contact precaution), by pathogen. CRE= Carbapenem-resistant Enterobacterales. MRSA= Methicillin-resistant *Staphylococcus aureus*.

# IX. Guidelines for economic evaluations and transmission models

**Table A14.** CHEERS 2022 Checklist

| **Topic** | **No.** | **Item** | **Location where item is reported** |
| --- | --- | --- | --- |
| **Title** |  |  |  |
|  | 1 | Identify the study as an economic evaluation and specify the interventions being compared. | Title, Page 2 |
| **Abstract** |  |  |  |
|  | 2 | Provide a structured summary that highlights context, key methods, results, and alternative analyses. | Abstract, Page 2 |
| **Introduction** |  |  |  |
| **Background and objectives** | 3 | Give the context for the study, the study question, and its practical relevance for decision making in policy or practice. | Introduction, Line 7 |
| **Methods** |  |  |  |
| **Health economic analysis plan** | 4 | Indicate whether a health economic analysis plan was developed and where available. | Methods, Line 5 |
| **Study population** | 5 | Describe characteristics of the study population (such as age range, demographics, socioeconomic, or clinical characteristics). | Methods, Line 10 |
| **Setting and location** | 6 | Provide relevant contextual information that may influence findings. | Methods, Third Paragraph |
| **Comparators** | 7 | Describe the interventions or strategies being compared and why chosen. | Methods, Fourth Paragraph |
| **Perspective** | 8 | State the perspective(s) adopted by the study and why chosen. | Methods, Fifth Paragraph |
| **Time horizon** | 9 | State the time horizon for the study and why appropriate. | Methods, Fifth Paragraph |
| **Discount rate** | 10 | Report the discount rate(s) and reason chosen. | Methods, Fifth Paragraph |
| **Selection of outcomes** | 11 | Describe what outcomes were used as the measure(s) of benefit(s) and harm(s). | Methods, Sixth Paragraph |
| **Measurement of outcomes** | 12 | Describe how outcomes used to capture benefit(s) and harm(s) were measured. | Methods, Sixth Paragraph |
| **Valuation of outcomes** | 13 | Describe the population and methods used to measure and value outcomes. | Methods, Sixth Paragraph |
| **Measurement and valuation of resources and costs** | 14 | Describe how costs were valued. | Not reported |
| **Currency, price date, and conversion** | 15 | Report the dates of the estimated resource quantities and unit costs, plus the currency and year of conversion. | Methods, Seventh Paragraph |
| **Rationale and description of model** | 16 | If modelling is used, describe in detail and why used. Report if the model is publicly available and where it can be accessed. | Methods, Eighth Paragraph |
| **Analytics and assumptions** | 17 | Describe any methods for analysing or statistically transforming data, any extrapolation methods, and approaches for validating any model used. | Methods , Last paragraph and Appendix |
| **Characterising heterogeneity** | 18 | Describe any methods used for estimating how the results of the study vary for subgroups. | Methods , Last paragraph |
| **Characterising distributional effects** | 19 | Describe how impacts are distributed across different individuals or adjustments made to reflect priority populations. | Methods , Last paragraph |
| **Characterising uncertainty** | 20 | Describe methods to characterise any sources of uncertainty in the analysis. | Not applicable |
| **Approach to engagement with patients and others affected by the study** | 21 | Describe any approaches to engage patients or service recipients, the general public, communities, or stakeholders (such as clinicians or payers) in the design of the study. | Appendix |
| **Results** |  |  |  |
| **Study parameters** | 22 | Report all analytic inputs (such as values, ranges, references) including uncertainty or distributional assumptions. | Results, first paragraph and Table |
| **Summary of main results** | 23 | Report the mean values for the main categories of costs and outcomes of interest and summarise them in the most appropriate overall measure. | Results, second paragraph |
| **Effect of uncertainty** | 24 | Describe how uncertainty about analytic judgments, inputs, or projections affect findings. Report the effect of choice of discount rate and time horizon, if applicable. | Results, third paragraph |
| **Effect of engagement with patients and others affected by the study** | 25 | Report on any difference patient/service recipient, general public, community, or stakeholder involvement made to the approach or findings of the study | Not reported |
| **Discussion** |  |  |  |
| **Study findings, limitations, generalisability, and current knowledge** | 26 | Report key findings, limitations, ethical or equity considerations not captured, and how these could affect patients, policy, or practice. | Discussion |
| **Other relevant information** |  |  |  |
| **Source of funding** | 27 | Describe how the study was funded and any role of the funder in the identification, design, conduct, and reporting of the analysis | End of manuscript |
| **Conflicts of interest** | 28 | Report authors conflicts of interest according to journal or International Committee of Medical Journal Editors requirements. | End of manuscript |

*From:* Husereau D, Drummond M, Augustovski F, et al. Consolidated Health Economic Evaluation Reporting Standards 2022 (CHEERS 2022) Explanation and Elaboration: A Report of the ISPOR CHEERS II Good Practices Task Force. Value Health 2022;25. <doi:10.1016/j.jval.2021.10.008>

# X. STROBE guidelines for observational studies

STROBE Statement—checklist of items that should be included in reports of observational studies

|  | Item No | Recommendation | Page  No |
| --- | --- | --- | --- |
| **Title and abstract** | 1 | (*a*) Indicate the study’s design with a commonly used term in the title or the abstract | 2 |
|  |  | (*b*) Provide in the abstract an informative and balanced summary of what was done and what was found | 2-3 |
| Introduction | | | |
| Background/rationale | 2 | Explain the scientific background and rationale for the investigation being reported | 4 |
| Objectives | 3 | State specific objectives, including any prespecified hypotheses | 4-5 |
| Methods | | | |
| Study design | 4 | Present key elements of study design early in the paper | 4-6 |
| Setting | 5 | Describe the setting, locations, and relevant dates, including periods of recruitment, exposure, follow-up, and data collection | 4-5 |
| Participants | 6 | (*a*) *Cohort study*—Give the eligibility criteria, and the sources and methods of selection of participants. Describe methods of follow-up  *Case-control study*—Give the eligibility criteria, and the sources and methods of case ascertainment and control selection. Give the rationale for the choice of cases and controls  *Cross-sectional study*—Give the eligibility criteria, and the sources and methods of selection of participants | 4-5 |
|  |  | (*b*) *Cohort study*—For matched studies, give matching criteria and number of exposed and unexposed  *Case-control study*—For matched studies, give matching criteria and the number of controls per case |  |
| Variables | 7 | Clearly define all outcomes, exposures, predictors, potential confounders, and effect modifiers. Give diagnostic criteria, if applicable | 4-5 |
| Data sources/ measurement | 8* | For each variable of interest, give sources of data and details of methods of assessment (measurement). Describe comparability of assessment methods if there is more than one group | *4-5* |
| Bias | 9 | Describe any efforts to address potential sources of bias | 4-6 |
| Study size | 10 | Explain how the study size was arrived at | 4-5 |
| Quantitative variables | 11 | Explain how quantitative variables were handled in the analyses. If applicable, describe which groupings were chosen and why | 4-5 |
| Statistical methods | 12 | (*a*) Describe all statistical methods, including those used to control for confounding | 4-6 |
|  |  | (*b*) Describe any methods used to examine subgroups and interactions | 4-5 |
|  |  | (*c*) Explain how missing data were addressed | 4-6 |
|  |  | (*d*) *Cohort study*—If applicable, explain how loss to follow-up was addressed  *Case-control study*—If applicable, explain how matching of cases and controls was addressed  *Cross-sectional study*—If applicable, describe analytical methods taking account of sampling strategy |  |
|  |  | (*e*) Describe any sensitivity analyses | 5-6 |

| Results | | | |
| --- | --- | --- | --- |
| Participants | 13* | (a) Report numbers of individuals at each stage of study—eg numbers potentially eligible, examined for eligibility, confirmed eligible, included in the study, completing follow-up, and analysed | 6-7 |
|  |  | (b) Give reasons for non-participation at each stage | 6-7 |
|  |  | (c) Consider use of a flow diagram |  |
| Descriptive data | 14* | (a) Give characteristics of study participants (eg demographic, clinical, social) and information on exposures and potential confounders | 6-7 |
|  |  | (b) Indicate number of participants with missing data for each variable of interest | 6-7 |
|  |  | (c) *Cohort study*—Summarise follow-up time (eg, average and total amount) |  |
| Outcome data | 15* | *Cohort study*—Report numbers of outcome events or summary measures over time |  |
|  |  | *Case-control study—*Report numbers in each exposure category, or summary measures of exposure |  |
|  |  | *Cross-sectional study—*Report numbers of outcome events or summary measures | *6-7* |
| Main results | 16 | (*a*) Give unadjusted estimates and, if applicable, confounder-adjusted estimates and their precision (eg, 95% confidence interval). Make clear which confounders were adjusted for and why they were included | 6-8 |
|  |  | (*b*) Report category boundaries when continuous variables were categorized | 6-8 |
|  |  | (*c*) If relevant, consider translating estimates of relative risk into absolute risk for a meaningful time period |  |
| Other analyses | 17 | Report other analyses done—eg analyses of subgroups and interactions, and sensitivity analyses | 7-8 |
| Discussion | | | |
| Key results | 18 | Summarise key results with reference to study objectives | 8 |
| Limitations | 19 | Discuss limitations of the study, taking into account sources of potential bias or imprecision. Discuss both direction and magnitude of any potential bias | 9 |
| Interpretation | 20 | Give a cautious overall interpretation of results considering objectives, limitations, multiplicity of analyses, results from similar studies, and other relevant evidence | 8-9 |
| Generalisability | 21 | Discuss the generalisability (external validity) of the study results | 8-9 |
| Other information | | | |
| Funding | 22 | Give the source of funding and the role of the funders for the present study and, if applicable, for the original study on which the present article is based | 2 |

*Give information separately for cases and controls in case-control studies and, if applicable, for exposed and unexposed groups in cohort and cross-sectional studies.

**Note:** An Explanation and Elaboration article discusses each checklist item and gives methodological background and published examples of transparent reporting. The STROBE checklist is best used in conjunction with this article (freely available on the Web sites of PLoS Medicine at http://www.plosmedicine.org/, Annals of Internal Medicine at http://www.annals.org/, and Epidemiology at http://www.epidem.com/). Information on the STROBE Initiative is available at www.strobe-statement.org.

# XI. Additional references

1. Allel K, Peters A, Haghparast-Bidgoli H, et al. Excess Burden of Antibiotic-Resistant Bloodstream Infections: Evidence from a Multicentre Retrospective Cohort Study in Chile, 2018-2022. *Available at SSRN 4676961* 2024.

2. Wertheim HF, Melles DC, Vos MC, et al. The role of nasal carriage in Staphylococcus aureus infections. *The Lancet infectious diseases* 2005; **5**(12): 751-62.

3. Allel K, Labarca J, Carvajal C, et al. Trends and socioeconomic, demographic, and environmental factors associated with antimicrobial resistance: a longitudinal analysis in 39 hospitals in Chile 2008–2017. *The Lancet Regional Health–Americas* 2023; **21**.

4. Safdar N, Bradley EA. The risk of infection after nasal colonization with Staphylococcus aureus. *The American journal of medicine* 2008; **121**(4): 310-5.

5. Araos R, Smith RM, Styczynski A, et al. High burden of intestinal colonization with antimicrobial-resistant bacteria in Chile: an antibiotic resistance in communities and hospitals (ARCH) study. *Clinical Infectious Diseases* 2023; **77**(Supplement_1): S75-S81.

6. Borer A, Saidel-Odes L, Eskira S, et al. Risk factors for developing clinical infection with carbapenem-resistant Klebsiella pneumoniae in hospital patients initially only colonized with carbapenem-resistant K pneumoniae. *American journal of infection control* 2012; **40**(5): 421-5.

7. Rao K, Patel A, Sun Y, et al. Risk factors for Klebsiella infections among hospitalized patients with preexisting colonization. *Msphere* 2021; **6**(3): e00132-21.

8. Shenoy ES, Paras ML, Noubary F, Walensky RP, Hooper DC. Natural history of colonization with methicillin-resistant Staphylococcus aureus (MRSA) and vancomycin-resistant Enterococcus (VRE): a systematic review. *BMC infectious diseases* 2014; **14**(1): 1-13.

9. Troché G, Toly L-M, Guibert M, Zazzo J-F. Detection and treatment of antibiotic-resistant bacterial carriage in a surgical intensive care unit: a 6-year prospective survey. *Infection Control & Hospital Epidemiology* 2005; **26**(2): 161-5.

10. Indicators O, Hagvísar O. Health at a glance 2019: OECD indicators. Average length of stay in hospitals: Paris: OECD Publishing; 2019.

11. Nielsen KL, Pedersen TM, Udekwu KI, et al. Fitness cost: a bacteriological explanation for the demise of the first international methicillin-resistant Staphylococcus aureus epidemic. *Journal of antimicrobial chemotherapy* 2012; **67**(6): 1325-32.

12. Hogea C, Van Effelterre T, Acosta C. A basic dynamic transmission model of Staphylococcus aureus in the US population. *Epidemiology & Infection* 2014; **142**(3): 468-78.

13. Balm MN, Lover AA, Salmon S, Tambyah PA, Fisher DA. Progression from new methicillin-resistant Staphylococcus aureus colonisation to infection: an observational study in a hospital cohort. *BMC infectious diseases* 2013; **13**: 1-8.

14. D'Agata EM, Webb GF, Horn MA, Moellering RC, Ruan S. Modeling the invasion of community-acquired methicillin-resistant Staphylococcus aureus into hospitals. *Clinical Infectious Diseases* 2009; **48**(3): 274-84.

15. Fishbain JT, Lee JC, Nguyen HD, et al. Nosocomial transmission of methicillin-resistant Staphylococcus aureus: a blinded study to establish baseline acquisition rates. *Infection Control & Hospital Epidemiology* 2003; **24**(6): 415-21.

16. Wangchinda W, Thamlikitkul V, Watcharasuwanseree S, Tangkoskul T. Active surveillance for carbapenem-resistant Enterobacterales (CRE) colonization and clinical course of CRE colonization among hospitalized patients at a University Hospital in Thailand. *Antibiotics* 2022; **11**(10): 1401.

17. Vanacker M, Lenuzza N, Rasigade J-P. The fitness cost of horizontally transferred and mutational antimicrobial resistance in Escherichia coli. *Frontiers in Microbiology* 2023; **14**.

18. Pérez-Galera S, Bravo-Ferrer JM, Paniagua M, et al. Risk factors for infections caused by carbapenem-resistant Enterobacterales: an international matched case-control-control study (EURECA). *Eclinicalmedicine* 2023; **57**.

19. Chen X, Wen X, Jiang Z, Yan Q. Prevalence and factors associated with carbapenem-resistant Enterobacterales (CRE) infection among hematological malignancies patients with CRE intestinal colonization. *Annals of Clinical Microbiology and Antimicrobials* 2023; **22**(1): 1-10.

20. Gomides MDA, Fontes AMdS, Silveira AOSM, Matoso DC, Ferreira AL, Sadoyama G. The importance of active surveillance of carbapenem-resistant Enterobacterales (CRE) in colonization rates in critically ill patients. *Plos one* 2022; **17**(1): e0262554.

21. Saidel-Odes L, Polachek H, Peled N, et al. A randomized, double-blind, placebo-controlled trial of selective digestive decontamination using oral gentamicin and oral polymyxin E for eradication of carbapenem-resistant Klebsiella pneumoniae carriage. *Infection Control & Hospital Epidemiology* 2012; **33**(1): 14-9.

22. Oren I, Sprecher H, Finkelstein R, et al. Eradication of carbapenem-resistant Enterobacteriaceae gastrointestinal colonization with nonabsorbable oral antibiotic treatment: a prospective controlled trial. *American journal of infection control* 2013; **41**(12): 1167-72.

23. Richter SS, Marchaim D. Screening for carbapenem-resistant Enterobacteriaceae: who, when, and how? *Virulence* 2017; **8**(4): 417-26.

24. Samra Z, Bahar J, Madar-Shapiro L, Aziz N, Israel S, Bishara J. Evaluation of CHROMagar KPC for rapid detection of carbapenem-resistant Enterobacteriaceae. *Journal of clinical microbiology* 2008; **46**(9): 3110-1.

25. Pournaras S, Zarkotou O, Poulou A, et al. A combined disk test for direct differentiation of carbapenemase-producing Enterobacteriaceae in surveillance rectal swabs. *Journal of clinical microbiology* 2013; **51**(9): 2986-90.

26. Vasoo S, Lolans K, Li H, Prabaker K, Hayden MK. Comparison of the CHROMagar™ KPC, Remel Spectra™ CRE, and a direct ertapenem disk method for the detection of KPC-producing Enterobacteriaceae from perirectal swabs. *Diagnostic microbiology and infectious disease* 2014; **78**(4): 356-9.

27. Hindiyeh M, Smollen G, Grossman Z, et al. Rapid detection of bla KPC carbapenemase genes by real-time PCR. *Journal of clinical microbiology* 2008; **46**(9): 2879-83.

28. Nahimana I, Francioli P, Blanc D. Evaluation of three chromogenic media (MRSA-ID, MRSA-Select and CHROMagar MRSA) and ORSAB for surveillance cultures of methicillin-resistant Staphylococcus aureus. *Clinical Microbiology and Infection* 2006; **12**(12): 1168-74.

29. Van Hal S, Stark D, Lockwood B, Marriott D, Harkness J. Methicillin-resistant Staphylococcus aureus (MRSA) detection: comparison of two molecular methods (IDI-MRSA PCR assay and GenoType MRSA Direct PCR assay) with three selective MRSA agars (MRSA ID, MRSA Select, and CHROMagar MRSA) for use with infection-control swabs. *Journal of clinical microbiology* 2007; **45**(8): 2486-90.

30. Perry JD, Davies A, Butterworth LA, Hopley AL, Nicholson A, Gould FK. Development and evaluation of a chromogenic agar medium for methicillin-resistant Staphylococcus aureus. *Journal of clinical microbiology* 2004; **42**(10): 4519-23.

31. Robotham JV, Graves N, Cookson BD, et al. Screening, isolation, and decolonisation strategies in the control of meticillin resistant Staphylococcus aureus in intensive care units: cost effectiveness evaluation. *Bmj* 2011; **343**.

32. Compernolle V, Verschraegen G, Claeys G. Combined use of Pastorex Staph-Plus and either of two new chromogenic agars, MRSA ID and CHROMagar MRSA, for detection of methicillin-resistant Staphylococcus aureus. *Journal of Clinical Microbiology* 2007; **45**(1): 154-8.

33. Malhotra-Kumar S, Haccuria K, Michiels M, et al. Current trends in rapid diagnostics for methicillin-resistant Staphylococcus aureus and glycopeptide-resistant enterococcus species. *Journal of clinical microbiology* 2008; **46**(5): 1577-87.

34. Harbarth S, Masuet-Aumatell C, Schrenzel J, et al. Evaluation of rapid screening and pre-emptive contact isolation for detecting and controlling methicillin-resistant Staphylococcus aureus in critical care: an interventional cohort study. *Critical care* 2006; **10**: 1-8.

35. Ho K-w, Ng W-t, Ip M, You JH. Active surveillance of carbapenem-resistant Enterobacteriaceae in intensive care units: Is it cost-effective in a nonendemic region? *American Journal of Infection Control* 2016; **44**(4): 394-9.

36. Dortet L, Bréchard L, Cuzon G, Poirel L, Nordmann P. Strategy for rapid detection of carbapenemase-producing Enterobacteriaceae. *Antimicrobial agents and chemotherapy* 2014; **58**(4): 2441-5.

37. Kypraios T, O'Neill PD, Huang SS, Rifas-Shiman SL, Cooper BS. Assessing the role of undetected colonization and isolation precautions in reducing methicillin-resistant Staphylococcus aureus transmission in intensive care units. *BMC infectious diseases* 2010; **10**: 1-10.

38. Ben-David D, Masarwa S, Adler A, Mishali H, Carmeli Y, Schwaber MJ. A national intervention to prevent the spread of carbapenem-resistant Enterobacteriaceae in Israeli post-acute care hospitals. *Infection Control & Hospital Epidemiology* 2014; **35**(7): 802-9.

39. You JH, Li H-k, Ip M. Surveillance-guided selective digestive decontamination of carbapenem-resistant Enterobacteriaceae in the intensive care unit: A cost-effectiveness analysis. *American Journal of Infection Control* 2018; **46**(3): 291-6.

40. Lübbert C, Faucheux S, Becker-Rux D, et al. Rapid emergence of secondary resistance to gentamicin and colistin following selective digestive decontamination in patients with KPC-2-producing Klebsiella pneumoniae: a single-centre experience. *International journal of antimicrobial agents* 2013; **42**(6): 565-70.

41. Zhang SX, Drews SJ, Tomassi J, Katz KC. Comparison of two versions of the IDI-MRSA assay using charcoal swabs for prospective nasal and nonnasal surveillance samples. *Journal of clinical microbiology* 2007; **45**(7): 2278-80.

42. Huletsky A, Huletsky A, Lebel P, et al. Identification of methicillin-resistant Staphylococcus aureus carriage in less than 1 hour during a hospital surveillance program. *Clinical Infectious Diseases* 2005; **40**(7): 976-81.

43. Rossney A, Herra C, Fitzgibbon M, Morgan P, Lawrence M, O’connell B. Evaluation of the IDI-MRSA assay on the SmartCycler real-time PCR platform for rapid detection of MRSA from screening specimens. *European Journal of Clinical Microbiology & Infectious Diseases* 2007; **26**: 459-66.

44. Paule SM, Hacek DM, Kufner B, et al. Performance of the BD GeneOhm methicillin-resistant Staphylococcus aureus test before and during high-volume clinical use. *Journal of clinical microbiology* 2007; **45**(9): 2993-8.

45. Bishop EJ, Grabsch EA, Ballard SA, et al. Concurrent analysis of nose and groin swab specimens by the IDI-MRSA PCR assay is comparable to analysis by individual-specimen PCR and routine culture assays for detection of colonization by methicillin-resistant Staphylococcus aureus. *Journal of Clinical Microbiology* 2006; **44**(8): 2904-8.

46. Rohr U, Mueller C, Wilhelm M, Muhr G, Gatermann S. Methicillin-resistant Staphylococcus aureus whole-body decolonization among hospitalized patients with variable site colonization by using mupirocin in combination with octenidine dihydrochloride. *Journal of Hospital Infection* 2003; **54**(4): 305-9.

47. van Rijen M, Bonten M, Wenzel R, Kluytmans J. Mupirocin ointment for preventing Staphylococcus aureus infections in nasal carriers. *Cochrane database of systematic reviews* 2008; (4).

48. Fondo Nacional de Salud. Aranceles mai 2023. <https://www.fonasa.cl/sites/fonasa/prestadores/modalidad-atencion-institucional#aranceles-mai-2023> (accessed 28 February 2024).

49. Central de abastacemiento (CENABAST) Chile. Reporte de compras historicas de CENABAST. 2023. <https://www.cenabast.cl/compras-cenabast/> (accessed 28 February 2024).

50. Mercado Publico. Orden de Compra. Nº5019-189-SE20 "INSUMOS DE LABORATORIO". 2024. <https://www.mercadopublico.cl/PurchaseOrder/Modules/PO/DetailsPurchaseOrder.aspx?qs=nvShVWA4/jRJQsD0aedHeg>== (accessed 6 March 2024).

51. Salary Expert. Average base salary of a registered nurse in Chile. 2024. <https://www.salaryexpert.com/salary/job/registered-nurse/chile> (accessed 5 March 2024).

52. Salary Expert. Average salary of a registered medical doctor in Chile. 2024. <https://www.salaryexpert.com/salary/job/doctor/chile#:~:text=758%20(CLP)%2Fyr&text=The%20average%20doctor%20gross%20salary,equivalent%20hourly%20rate%20of%20%2433.861>. (accessed 5 March 2024).

53. Gold MR, Franks P, McCoy KI, Fryback DG. Toward consistency in cost-utility analyses: using national measures to create condition-specific values. *Medical care* 1998: 778-92.

54. Ridley S, Morris S. Cost effectiveness of adult intensive care in the UK. *Anaesthesia* 2007; **62**(6): 547-54.

55. Tengs TO, Wallace A. One thousand health-related quality-of-life estimates. *Medical care* 2000: 583-637.

56. Gebretekle GB, Mariam DH, Mac S, et al. Cost–utility analysis of antimicrobial stewardship programme at a tertiary teaching hospital in Ethiopia. *BMJ open* 2021; **11**(12): e047515.

57. Honselmann KC, Buthut F, Heuwer B, et al. Long-term mortality and quality of life in intensive care patients treated for pneumonia and/or sepsis: Predictors of mortality and quality of life in patients with sepsis/pneumonia. *Journal of critical care* 2015; **30**(4): 721-6.

58. Wolbers M, Koller MT, Witteman JC, Steyerberg EW. Prognostic models with competing risks: methods and application to coronary risk prediction. *Epidemiology* 2009: 555-61.
